# Variant Surface Glycoprotein gene repertoires in *Trypanosoma brucei *have diverged to become strain-specific

**DOI:** 10.1186/1471-2164-8-234

**Published:** 2007-07-13

**Authors:** O Clyde Hutchinson, Kim Picozzi, Nicola G Jones, Helen Mott, Reuben Sharma, Susan C Welburn, Mark Carrington

**Affiliations:** 1 Department of Biochemistry, 80 Tennis Court Road, Cambridge, CB2 1GA, UK; 2 Centre for Tropical Veterinary Medicine, University of Edinburgh, Easter Bush Veterinary Centre, Roslin, Midlothian, EH25 9RG, UK; 3 Institute of Zoology, Zoological Society of London, Regents Park, London, NW1 4RY, UK; 4 Faculty of Veterinary Medicine, Universiti Putra Malaysia, 43400 UPM, Serdang, Selangor, Malaysia

## Abstract

**Background:**

In a mammalian host, the cell surface of African trypanosomes is protected by a monolayer of a single variant surface glycoprotein (VSG). The VSG is central to antigenic variation; one VSG gene is expressed at any one time and there is a low frequency stochastic switch to expression of a different VSG gene. The genome of *Trypanosoma brucei *contains a repertoire of > 1000 VSG sequences. The degree of conservation of the genomic VSG repertoire in different strains has not been investigated in detail.

**Results:**

Eighteen expressed VSGs from Ugandan isolates were compared with homologues (> 40 % sequence identity) in the two available *T. brucei *genome sequences. Fourteen homologues were present in the genome of *Trypanosoma brucei brucei *TREU927 from Kenya and fourteen in the genome of *T. b. gambiense *Dal972 from Cote d'Ivoire. The Ugandan VSGs averaged 71% and 73 % identity to homologues in *T. b. brucei *and *T. b. gambiense *respectively. The sequence divergence between homologous VSGs from the three different strains was not random but was more prevalent in the parts of the VSG believed to interact with the host immune system on the living trypanosome.

**Conclusion:**

It is probable that the VSG repertoires in the different isolates contain many common VSG genes. The location of divergence between VSGs is consistent with selection for strain-specific VSG repertoires, possibly to allow superinfection of an animal by a second strain. A consequence of strain-specific VSG repertoires is that any vaccine based on large numbers of VSGs from a single strain will only provide partial protection against other strains.

## Background

*Trypanosoma brucei *infects a wide range of larger mammals across sub-Saharan Africa. *T. brucei *and some other species of the African trypanosomes have evolved a population survival strategy in the mammalian host based on the generation and clonal expansion of new antigenic variants at a rate fast enough to prevent recognition of the whole population by the host immune response. Most indigenous wild host species are tolerant to trypanosome infections exhibiting low parasitaemias with limited display of patent symptoms [1]. Similar tolerance has been selected for in some indigenous breeds of cattle [2] although infection reduces productivity [3]. In contrast, indigenous breeds of cattle from outside the range of the tsetse fly (the insect vector) and introduced animals such as Western European dairy breeds, horses and dogs, are susceptible and can have severe clinical symptoms [2,4]. Although *T. brucei *can both establish and maintain an infection in a mammalian host, it is unclear whether an infection acquired early in life persists for the lifetime of the host, say 3 to 15 years, or whether the host is repeatedly superinfected by an increasing number of strains as a result of constant exposure to infected tsetse flies.

Antigenic variation in trypanosomes is dependent on a protective protein coat that covers the entire surface of the trypanosome [5]. The coat is composed of a single protein, the variant surface glycoprotein (VSG), that protects the plasma membrane from complement and invariant cell surface proteins from recognition by host immunoglobulins [6,7]. An infecting population expresses a series of VSGs from a large reservoir of VSG sequences in the genome [8,9]. Different VSGs are antigenically distinct due to extreme variation in sequence but have a conserved structure, presumably necessary for their function as a protective barrier [10,11]. *T. brucei *VSGs are composed of a combination of one N-terminal domain of ~340 residues and one or two C-terminal domains of 30 to 50 residues each [12]. The N-terminal domains have been categorized into three types, A, B and C, according to two features of the primary structure: the location of conserved cysteine residues and the presence of a heptad repeat in a region known to form a coiled coil [10,12]. The C-terminal domains have been divided into six types, 1 to 6, based on the location of conserved cysteine residues and the sequence of the C-terminal glycosylphosphatidylinositol-anchor addition signal [9,12]. There appears to be no restriction on domain combinations and similar N-terminal domains have been found with different C-terminal domains [12,13].

The structure of the genomic reservoir of VSGs was determined as part of the genome project [9]. There are between 1000 and 2000 potential VSG sequences in the genome; however, only 7 % encode functional VSG open reading frames (ORFs) and of the remainder 9 % encode an ORF for a VSG with atypical primary structure; 62 % are disrupted VSG ORFs containing frame shifts and/or stop codons and the remainder are fragmentary VSG ORFs. Around 10 % of the VSG sequences lie at telomeres of large, intermediate and mini-chromosomes but the majority are present in subtelomeric tandem arrays [9,14] The telomeric VSG genes are under represented in the genome sequence and the final percentage encoding functional VSG genes will probably increase to 10 to 15 % as telomeres are sequenced. The genome is diploid with the exception of the sub-telomeric and telomeric VSG sequence arrays which differ between homologous chromosomes to such a great extent that the arrays are effectively haploid [9].

The rate of switching expression from one VSG to another is < 0.01 per cell generation, and so most trypanosomes are eventually cleared by the host immune system as they do not switch VSGs [15]. However, the rate of switching is high enough to maintain the infection provided the reservoir of immunologically novel VSGs is not exhausted. Thus, the long term persistence of an infection will be dependent on the size of the utilisable VSG reservoir. Given the large number of potential VSG sequence donors in the genome [9], the potential number of different VSGs may be thousands. More than 100 VSGs were serially expressed by a single *T. brucei *clone [16]. The only reported experiment to address exhaustion of the VSG repertoire used the related species *T. vivax *and found that clonally infected goats eventually self cured after one to two years [17].

Some VSGs are present in the genome as multiple copy families of closely related sequences [18]. However, the majority of genomic VSG sequences are single copy genes distantly related to other VSGs [9]. It is not known whether VSG sequence repertoires are expanding and/or diverging. Here, an analysis of eighteen expressed VSG genes is presented. The VSGs are all derived from field samples of *T. brucei *collected in the Tororo District of Uganda. The VSGs contain the same primary structural features as found in previously characterised VSGs. Close homologues of fourteen of the VSGs are present in the genome sequence of *T. b. brucei *TREU927 isolated in Kiboko, Kenya and equally close homologues of fourteen are present in the genome sequence of *T. b. gambiense *Dal972 isolated in Daloa, Côte d'Ivoire, West Africa. Comparisons of three homologues from each of the isolates showed that sequence divergence between homologous VSGs is concentrated in regions of the gene encoding the part of the N-terminal domain involved in direct interactions with host immune system.

## Results

Eighteen novel VSG cDNA sequences were obtained from eight field isolates from zebu cattle or pigs; twelve were *Trypanosoma brucei brucei *and six were the human-infective *T. b. rhodesiense*. All the isolates were originally collected in the Tororo district of Uganda in an area where *T. b. rhodesiense *is endemic and hundreds of kilometers from the region where the second human infective subspecies *T. b. gambiense *is endemic in north west Uganda [19] (see map in Additional File 1). The identifiers and database accession numbers for each of the VSGs described in this paper are given in Table 1.

**Table 1:** Comparison of VSGs from field isolates with VSG sequences present in the genome of *T. brucei brucei *TREU927 and *T. brucei gambiense *Dal972

Tororo VSG	ssp.	domains	Tbb 927 VSG with greatest identity	genomic VSG intact?	genomic sequence complete?	endpoint(s) of identity within ORF?	Tbg Dal VSG with greatest identity	genomic VSG intact?	genomic sequence complete?	endpoint(s) of identity within ORF?	Tororo VSG accession number
VSG Ako 1	Tbr	B1	contig 11608	No	partial	?	gamb276c12.p1k_3	No	Yes	CTD	AJ937323
VSG Ako 2	Tbb	B1	Tb11.24.0011	No	Yes	CTD	gamb843f08.p1k	No	Yes	CTD	AJ937324
VSG Bug 1	Tbb	A3	Tb927.5.190	No	Yes	SS CTD	gamb1660g03.q1k H	No	Yes	SS CTD	AJ560648
VSG Bug 2	Tbb	B2	-	-	-	-	gamb856d03.q1k_1	No	Yes	CTD	AJ937318
VSG Buteba 1	Tbb	B2	Tb10.v04.0152	No	Yes	CTD	gamb333c09.p1k H	-	partial	?	AJ549081
VSG Buteba 2	Tbb	A2	-	-	-	-	gamb1441g08.p1k H	No	partial	?	AJ937312
VSG Buteba 3	Tbb	B1	Tb09.142.0130	No	Yes	CTD	gamb1420g06.q1k H	No	Yes	CTD	AJ937316
VSG Buteba 4	Tbb	A2	Tb927.3.390	No	Yes	CTD	chr3	No	Yes	CTD	AJ937321
VSG Buteba 6	Tbb	B1	Tb927_11_02_v4	No	Yes	CTD	-	-	-	-	AJ937329
VSG Buw 1	Tbb	B2	Tb09.244.0090	No	Yes	CTD	gamb217f04.p1k	No	Yes	SS CTD	AJ937326
VSG Buw 2	Tbr	A2	Tb05.26C7.320	No	Yes	CTD	-	-	-	-	AJ937328
VSG Do 1	Tbr	B2	Tb09.244.1110	No	Yes	SS hinge	gamb261a02.p1k	No	Yes	SS hinge	AJ937315
VSG Do 2	Tbr	B1	tryp_XI-346e09.p1k	No	partial	?	-	-	-	-	AJ937325
VSG Kinu 1	Tbb	A2	Tb09.244.0250	Yes	Yes	outside ORF	gamb606g06.q1k H	-	partial	?	AJ937313
VSG Maw-ero 1	Tbr	B1	-	-	-	-	gamb1327e07.q1k	No	Yes	SS	AJ937314
VSG Mul 1	Tbr	B2	-	-	-	-	-	-	-	-	AJ937317
VSG Mul 3	Tbb	B2	Tb10.v4.0093	No	Yes	CTD	gamb86e09.p1k H	No	partial	?	AJ937320
VSG Mul 4	Tbb	B2	Tb09.244.1190	No	Yes	CTD	gamb248h03.p1k H	No	Yes	CTD	AJ937327

Most of the VSGs expressed by field isolates are present in the genome sequences of both *T. brucei brucei *TREU927 and *T. brucei gambiense *Dal972. The table shows the name of the VSG from the Tororo district, whether it is from *T. b. brucei *(Tbb) or *T. b. rhodesiense *(Tbr) and the domain type combination. The most similar VSG in the genome sequences of *T. b. brucei *TREU927 and *T. b. gambiense *Dal972 is then given, the cut off for inclusion was sequence identity of > 40 % over the whole of the VSG. In some cases the sequence was from manual assembly of individual sequencing runs into contigs and these are available in the Supplementary Material and are marked H after the identifier. The 'intact VSG' column indicates whether the VSG sequences in the genome encode a potentially functional VSG ORF (Berriman et al. 2005). The 'database entry complete' column indicates whether the sequence contig or sequence read spanned the entire potential ORF. The endpoints of identity column indicates where, within the VSG amino acid sequence, the high percentage identity ended: SS, N-terminal signal sequence; hinge, linker between the two domains; CTD, C-terminal domain.

### The field isolate VSGs have typical domain structures

The Tororo VSGs was analysed to determine which N-terminal and C-terminal domain types they contained. The identification of domain types was based on the location of conserved cysteine residues [9,12]. All the VSGs from Tororo contained a typical VSG domain structure and fell within previously characterized VSG types. N-terminal domain types A and B and C-terminal domain types 1 and 2 predominated (Table 1). The same is true for the collection of expressed VSGs previously sequenced, for example [12], and for the VSG sequences present in the *T. b. brucei *TREU927 genome [9]. Thus, the Tororo VSGs have typical VSG primary structures.

### Close homologues of the Tororo VSGs are present in both East and West African *T. brucei *genomes

The degree to which *T. brucei *isolates from different geographic regions share a common VSG repertoire is not known. The Tororo VSG sequences were used to identify any homologues in the genome sequences of *T. b. brucei *TREU927 (*Tbb*927) [9], isolated from a tsetse fly in Kiboko, Kenya [20,21] and the unfinished genome sequence of *T. b. gambiense *Dal972 clone 1 MHOM/CI/86/DAL972 (*Tbg*Dal) isolated in Daloa, Côte d'Ivoire, West Africa [22] (see map in the Additional File). For *T. b. brucei *TREU927, the chromosome assemblies, genome survey sequence assemblies and individual sequence reads were screened and for *T. b. gambiense *Dal972, both contigs and individual sequence reads were screened.

'Closely related' was arbitrarily defined as two VSG homologues having greater than 40 % amino acid sequence identity. Seventeen out of eighteen Tororo VSG had a closely related VSG sequence in either one or both of the *Tbb*927 or *Tbg*Dal genome sequences; fourteen in the *Tbb*927 genome and fourteen in the *Tbg*Dal genome (Table 1 and more information about the VSG homologues identified in the genome is in the Additional File). The twenty eight VSG sequences identified in the two genome sequences were examined. In all but one case the genomic copy encoded an incomplete VSG open reading frame (ORF), either with a frame shift, and/or a premature stop codon and/or the absence of a contiguously encoded C-terminal domain. The small number of intact VSG ORFs (1/28) is a reflection of the low percentage of intact VSG ORFs in the *Tbb*927 genome [9,14]. The Tororo VSGs had a single closely related homologue in the *Tbb*927 or *Tbg*Dal genome sequences and were not part of a large VSG multigene family, the next closest homologue having less than 40 % amino acid sequence identity. There was one possible exception that was difficult to analyse due to partial sequence in the genome database (data not shown). This result was unexpected as although 60 % of VSGs are single copy genes families the remaining 40 % are members of multigene families [9] (L Marcello and D. Barry, submitted). The Tororo VSGs were represented in each of the two genome sequence databases at a frequency of 78 % (14/18). This provides an indication that the genome of the Tororo isolates contains many VSG genes in common with the both the *Tbb*927 and *Tbg*Dal genomes. Using a *chi *squared test there is a 95 % probability that the Tororo isolates and each of the genome strains have greater than 58 % of VSG genes in common. Thus, the VSG repertoires of the Tororo isolates and each of the *Tbb*927 and *Tbg*Dal genomes are probably largely common.

Of the twenty eight genomic VSG sequences, two from *Tbb*927 and four from *Tbg*Dal were incomplete as the end of the available genomic sequence (contig or gel read) fell within the homologous Tororo VSG ORF (Table 1). The remaining twenty two genomic VSG sequences were used to investigate the extent of the sequence identity with the Tororo VSGs. An end point of sequence identity was apparent as a sudden decrease in sequence identity to background levels. In all cases (22/22), the identity extended for the entire region encoding the mature VSG N-terminal domain. In the majority (16/22), the 5' endpoint of identity occurred outside the VSG ORF and in the remainder (6/22) the sequence identity ended within the region encoding the N-terminal signal sequence. The 3' endpoint of sequence identity usually occurred within the region encoding the C-terminal domain (18/22), occasionally in the hinge between the two domains (2/22) or outside the region encoding the ORF (2/22). Thus, the genomic VSG sequences shared identity with the expressed VSGs over a region encoding the entire N-terminal domain but in nearly all cases this did not extend to include the complete C-terminal domain.

### The Ugandan Tororo VSGs have diverged to a similar extent from the VSGs present in the East African *T. b. brucei *TREU927 and West African *T. b. gambiense Dal972 *genomes

The amino acid sequences of the N-terminal domains were used to measure the similarity between the Tororo sequences and the genomic VSGs. The reading frame of some of the genomic VSG sequences was adjusted to maximize the amino acid sequence identity of the genomic VSG with the Tororo VSG by permitting shifting from one frame to another. Of the twenty eight genomic VSG sequences originally identified, twenty four encoded a complete N-terminal domain (Table 2); the remaining four were incomplete due to an end in the available genome sequence as opposed to a loss of identity (Table 2). Of the twenty four VSG N-terminal domain sequences identified from the genomes, half (12/24) encoding an intact and apparently functional VSG N-terminal domain without frame shifts or premature stop codons.

**Table 2:** Comparison of VSG N-terminal domains of field VSGs with genomic VSG sequences

database name	Tbb gene with greatest identity	NTD ORF intact?	identity (%)	Tbg gene with greatest identity	NTD ORF intact?	identity (%)	difference in % identities
VSG Ako 1	contig 11608	No	73	gamb276c12.p1k_3	Yes	84	11
VSG Ako 2	Tb11.24.0011	Yes	79	gamb843f08.p1k	No	79	0
VSG Bug 1	Tb927.5.190	Yes	58	gamb1660g03.q1k H	Yes	50	8
VSG Bug 2	-	-	-	gamb856d03.q1k_1	Yes	93	
VSG Buteba 1	Tb10.v04.0152	Yes	69	gamb333c09.p1k H	partial	67	2
VSG Buteba 2	-	-	-	gamb1441g08.p1k H	partial	84	
VSG Buteba 3	Tb09.142.0130	No	84	gamb1420g06.q1k H	Yes	92	8
VSG Buteba 4	Tb927.3.390	No	67	chr3	No	74	7
VSG Buteba 6	Tb927_11_02_v4	Yes	46	-	-	-	
VSG Buw 1	Tb09.244.0090	No	80	gamb217f04.p1k	No	69	11
VSG Buw 2	Tb05.26C7.320	No	74	-	-	-	
VSG Do 1	Tb09.244.1110	Yes	45	gamb261a02.p1k	Yes	44	1
VSG Do 2	tryp_XI-346e09.p1k	No	64	-	-	-	
VSG Kinu 1	Tb09.244.0250	Yes	95	gamb606g06.q1k H	-	93	2
VSG Maw-ero 1	-	-	-	gamb1327e07.q1k	partial	42	
VSG Mul 1	-	-	-	-	-	-	
VSG Mul 3	Tb10.v4.0093	No	83	gamb86e09.p1k H	No	85	2
VSG Mul 4	Tb09.244.1190	Yes	80	gamb248h03.p1k H	No	72	8

Average			71			73	

The N-terminal domains of the VSGs expressed by field isolates have similar percentage identities to homologues from *T. b. brucei *as to homologues from *T. b. gambiense*. The 'NTD ORF intact?' column indicates whether the genomic homologue encoded an N-terminal domain that was apparently functional and the percentage identity is over the whole N-terminal domain or all the available sequence (see Supplementary Material).

The percentage identity between the amino acid sequences of the N-terminal domains of the Tororo VSGs and the closely related VSGs from either of the two genome sequences was determined by pairwise comparisons (see the Additional File for the sequences). Twenty eight pairwise comparisons were made; twenty four comparisons between Tororo VSGs and the complete N-terminal domains identified in one or other of the genomes, and four partial sequences from one or other of the genomes were included as each encoded more than half a VSG N-terminal domain.

Identity varied from the arbitrary lower cut off of 40 % up to 95 %; the majority (16/28) were more than 70 % identical and the average identity was 71 % for comparisons between the Tororo VSGs and *Tbb*927 homologues and 73 % for comparisons between the Tororo VSGs and the *Tbg*Dal972 homologues (Table 2). A second observation from this analysis is that the percentage identity between any one Tororo VSG and each of the two genomic VSGs tended to be similar, for example the identities (Tororo against *Tbb*927and Tororo against *Tbg*Dal) for VSG Buteba 4 are 67 % and 74 % and for VSG Buteba 3 are 84 % and 92 %. The similarity in rate of divergence is shown as the difference between the two percentage identities in Table 2.

### The divergence between some homologous VSGs is consistent with selection for antigenically novel VSGs

**Figure 1:**
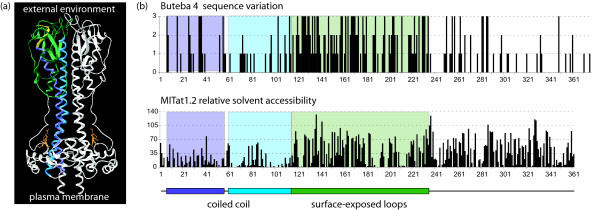
Location of sequence divergence and solvent accessibility in the primary and tertiary structure of VSG type A N-terminal domains. (a) The tertiary structure of a VSG N-terminal domain dimer with the tertiary structure features coloured in one monomer. The N-terminus is in yellow, the descending alpha helix of the long coiled coil is purple and the ascending helix blue; the surface loops at the top of the VSG are in green. (b) The colours are then used to highlight the same regions in the primary structure in plots of sequence variation (top) between VSG Buteba 4 and homologues from *T. b. brucei *and *T. b. gambiense *and below is shown the calculated solvent accessibility for the structurally related VSG MITat1.2.

It was possible to identify six Tororo VSGs that had homologues in both *Tbb*927and *Tbg*Dal with > 60 % identity in the N-terminal domain (Table 3). These six VSGs were used for three way comparisons of amino acid sequence (Alignments are in the Additional File). Of the six, VSG Buteba 4 was chosen for detailed analysis as it was the only one with a type A N-terminal domain which allowed a more certain structure-based alignment and comparison with the known structure of VSG MITat1.2 [10,23]. The VSG Buteba 4 N-terminal domain sequence was plotted against an arbitrary scale of the degree of variation at individual positions in the alignment of the three homologues (Figure 1). In addition, the solvent accessibility of individual residues in VSG MITat1.2 (PDB 1VSG) was calculated to provide a guide to the accessibility of residues in the Buteba 4 homologues (Figure 1). The divergence in sequence of the VSGs was concentrated in the region that was both solvent accessible and at the top of the VSG (green in Figure 1), this part of the VSG is believed to provide the primary targets of host immunoglobulins on living trypanosomes [24]. The second region of the VSG that is solvent accessible (residues 260 to 340) is at the base of the structure is not accessible to antibodies on the living trypanosome [24] and has not diverged as much is sequence.

**Table 3:** Percentage identities between the N-terminal domains of six triplets of VSGs used to analyse the location of sequence divergence in the primary and tertiary structure

		identity (%)			
		
Tororo VSG	NTD type	Tororo v 927	Tororo v Tbg	927 v Tbg	average
Buteba 3	B	84	92	82	86
Ako 2	B	79	79	87	82
Mul 4	B	80	82	79	80
Ako 1	B	73	84	71	76
Buw 1	B	80	69	78	76
Buteba 4	A	61	74	68	68

average		76	80	78	78

**Figure 2:**
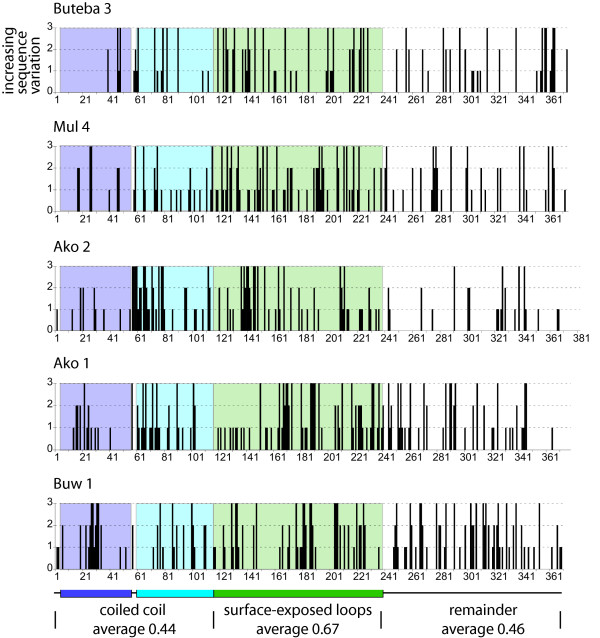
The location of sequence divergence five sets of three VSG type B in N-terminal domains. The descending alpha helix of the long coiled coil is purple and the ascending helix blue; the surface loops at the top of the VSG are in green. The colours are then used to highlight the same regions in the primary structure in plots of sequence variation. The average variation at each residue for the three different structural components of the VSG is shown below the primary structure representation.

A similar analysis was performed for the other five triplets of related VSGs (Table 3 and Figure 2). These five VSGs all have type B N-terminal domains and although there is firm evidence that they adopt a similar tertiary structure to type A VSGs [25] they are less well characterized. The analysis showed that the differences between the members of each triplet were more evenly distributed throughout the primary structure than for the Buteba 4 family. The average variation at each residue was calculated for the three structural parts of the VSG: the N-terminal coiled coil, the surface-exposed loops and the remained of the N-terminal domain. There was more variation per residue on average in the surface-exposed loops (0.67) then in either the coiled coil (0.44) or in the remainder of the domain (0.46) (Figure 2).

## Discussion

The main observations in this paper are: (i) the majority (17/18) of field isolates of *T. b. brucei *and *T. b. rhodesiense *from long-term infected domestic animals in Tororo in south eastern Uganda expressed VSGs that have close homologues (> 40 % amino acid sequence identity) in one or both of the available *T. b. brucei *TREU927 (*Tbb*927) and *T. b. gambiense *Dal972 (*Tbg*Dal) genome databases. (ii) The Tororo VSGs have diverged to a similar extent from homologues in the *Tbb*927 and *Tbg*Dal genomes despite the geographic and genetic separation of the *T. b. gambiense *isolate. (iii) Comparison of the Tororo VSGs with the homologues in both *Tbb*927 and *Tbg*Dal genomes indicates the diverged sequence is concentrated in the parts of the VSG that are predicted to be recognized by immunoglobulins on the living trypanosome.

Two observations made in the course of the comparison of the Tororo VSGs with the two genome sequences are relevant to the origin and maintenance of VSG repertoires in the *T. brucei *genome. First, most of the Tororo VSGs were represented in both the East African *Tbb*927 and the West African *Tbg*Dal genome sequences. This indicates that the current VSG repertoires in the three geographic locations evolved from a common ancestral repertoire. The overlap in repertoires reported here is likely to be less than the real overlap as VSG sequences are missing from both the *Tbb*927 and *Tbg*Dal genome databases due to under representation of mini-chromosomes and telomeres in the *Tbb*927 genome database [9] and possibly under representation in the *Tbg*Dal whole genome shotgun sequences. Second, the Tororo VSGs have diverged to a similar extent from the homologous VSGs in each of the *Tbb*927 and *Tbg*Dal genomes. This observation was surprising as *T. b. gambiense *has diverged from East African *T. brucei *ssp. and is distinguishable by a range of genetic markers [26], reviewed in [27]. This greater divergence of *T. b. gambiense *is not visible as a greater divergence of VSG sequences in the *Tbg*Dal genome. In addition, West African *T. b. gambiense *produces a different disease pathology to East African *T. b. rhodesiense *in humans, the data here hint that this difference is not caused by or reflected in differences in the VSG repertoire but the number of sequences analysed in insufficient to draw any conclusions.

Approximately 60 % of VSGs are encoded by single copy genes in the *Tbb*927 genome [9] (L Marcello and D. Barry, personal communication). So, the finding that nearly all the Tororo VSGs had single copy homologues in the *Tbb*927 and *Tbg*Dal genomes was unexpected. It possible that more homologues will be identified as VSG multicopy gene family members as more minichromosome and telomeric sequence becomes available. However, another possibility is that the VSGs were isolated from long term infected domestic animals and were represented by single copy VSG genes as they are expressed late in an infection [8,28].

Genetic exchange within a population and particularly meiotic recombination has the effect of homogenizing gene sequences. The ancestral VSG repertoire was presumably a product of genetic exchange within *T. brucei*. The subsequent divergence of VSG repertoires could have arisen if the trypanosomes from the three locations, Kenya, Uganda and Cote d'Ivoire, ceased meiotic genetic exchange of telomeric and sub-telomeric VSG genes at approximately the same time without affecting genetic exchange of other genes in the central parts of chromosomes which undoubtedly continues [29,30]. The model that VSGs are excluded from meiotic recombination suggests that subsequent to the appearance of the VSG repertoire an evolutionary step occurred that blocked meiotic crossovers occurring within the subtelomeric arrays of VSG genes so that reciprocal exchange between VSG arrays became rare, possibly through the failure of the arrays to pair in meiotic prophase. It is important to note that a failure to pair homologues during meiosis would not affect the VSG gene conversion events integral to antigenic variation during mitotic cell cycles. This latter model is consistent with the finding that the sub-telomeric tandem arrays of VSG genes are so different as to be considered haploid in contrast to the remainder of the chromosomes which are diploid [9] and that much of the difference in size between homologues can be explained by differences in the size of VSG gene tandem arrays [31]. The haploid VSG arrays permit a larger VSG repertoire per diploid genome and the exclusion of the arrays from genetic exchange would favour VSG divergence in different strains. A second possibility is that the cessation of genetic exchange between the West and East African isolates is explained by geographic separation. A similar explanation for the divergence of the East African isolates is more problematic but could have resulted from an unidentified ecological change imposing a barrier to free movement of infected mammals and/or infected tsetse flies. In contrast to VSGs, there is very little amino acid sequence variation in genes from the central parts of chromosomes. In a comparison of *Tbg*Dal with *Tbb*927 using an array of twenty genes in the central part of chromosome 2 all twenty *Tbb*927 genes had > 99 % amino acid sequence identity with the *Tbg*Dal genes (data not shown).

The most likely selection pressure for the divergence of individual VSGs in different strains is to favour superinfection of a host by a second trypanosome strain. If this is the case then the divergence between homologous VSG should be sufficient to produce antigenically distinct trypanosomes. How different do two VSGs have to be to produce antigenically non-cross reactive trypanosomes? This has only been investigated once through a serendipitous experiment in which trypanosomes expressing the VSG AnTat1.1b switched to the VSG AnTat1.10. Trypanosomes expressing VSG AnTat1.10 were protected from antibodies to VSG AnTat1.1b [32] yet the two VSG N-terminal domains have 69 % identity and the value rises to 74 % if an insertion in the AnTat1.10 sequence is excluded from the comparison (see the Additional File). The identity between the Tororo and genomic VSG N-terminal domains had a range of 61 to 92 %, so it is likely that many of the VSG homologues have evolved to be antigenically distinct when present on the trypanosome surface.

The observed difference in the rate of divergence of different VSGs (Tables 2 and 3) is consistent with earlier observations on the variation in VSGs in different stocks [33-35]. Experiments based on immunological cross reactivity of rabbit antibodies present after infection with a trypanosome clone [33] concluded that each strain had a characteristic set of VSGs expressed early in an infection and a limited number were shared between isolates. Experiments based on nucleic acid hybridisation [34,35] showed that some VSGs were present in all stocks, others in some stocks and some that were stock-specific. The problem in interpreting these results is that it is difficult to connect nucleic acid hybridisation with immunological cross-reactivity. However, the differences in VSG repertoires found are consistent with different rates of divergence observed here where some VSGs have diverged to become strain-specific whereas others are still recognisable as closely-related homologues.

The sequence divergence between the two AnTat VSGs described above is mostly in the surface-exposed loops of the VSG (green in Figure 1) and in the long coiled coil (purple and blue in Figure 1). The surface loops probably contain the B-cell epitopes [24] and the coiled coil may contain T-cell epitopes [10,18,36]. A similar pattern of sequence divergence has been reported for the eight members of the VSG MITat1.4 gene family present in a single genome from the *T. b. brucei *Lister 427 isolate [18]. It was proposed that the divergence of VSG family members provided a reservoir of sequences for the combinatorial production of novel mosaic VSGs. However, unlike the large VSG MITat1.4 gene family, nearly all the genomic VSG sequences identified here are single copy. Thus, it is unlikely that the sequence divergence observed between strains is due to sampling members of a multigene family but represents differences between homologues in different strains.

In the comparisons between the Tororo and *Tbb927 *and *TbgDal *genomic VSGs, the divergence was more prevalent in the surface-exposed loops but also occurred elsewhere (Figures 1 and 2). The regions of variation in the coiled coil are not solvent exposed (Figure 1) and although the divergence may be simply sequence drift, it is possible that the variable regions are involved in a more subtle interaction with the host T-cell response. Overall, the sequence divergence in the VSG N-terminal domains is consistent with selection for the generation of antigenically novel VSGs and that each strain has evolved or is evolving its own repertoire of VSGs. Such a divergence in VSG repertoires would allow superinfection of hosts by different strains and possibly a partially immune host by the same strain. A consequences would be that any vaccine based on a large number of VSGs from a single strain is unlikely to offer complete protection against a different strain.

A second possibility to explain the higher prevalence of sequence variation in the surfaceloops is that that region of the VSG polypeptide is more tolerant of sequence variation than the remainder of the VSG polypeptide. However, the ratio of synonymous to non-synonymous changes to codons was the same in the coiled coil and in the surface loops providing evidence that both structural elements have a roughly equal tolerence for mutation overall (data not shown). This observation is consistant with the model that the whole VSG structure is tolerant of sequence variation, as is witnessed by the VSGs sequences present in the genome, and different VSGs can vary over their entire amino acid sequence.

## Conclusion

Most of the expressed VSGs from Tororo had closely related homologues in the *Tbb*927 and *Tbg*Dal genomes indicating that genomic VSG repertoires probably contain a common set of VSG homologues. The Ugandan Tororo VSGs had diverged to a similar extent from homologues in the East and West African isolates indicating that the divergence probably started before the geographic dispersal of the strains and may indicate a reduced genetic exchange between VSG arrays. The location of divergence between VSG homologues was not random but was more prevalent in positions consistent with selection for the production of antigenically novel VSGs and thus strain-specific VSG repertoires. A consequence of strain-specific VSG repertoires is that any vaccine based on large numbers of VSGs from a single strain would only provide partial protection against other strains.

## Methods

*Trypanosoma brucei *isolates were collected in the Tororo district of Uganda [37]. All isolates were rapidly passaged twice through mice prior to import in order to comply with UK Government importation regulations.

RNA was prepared using Qiagen RNAeasy mini kits according to the manufacturer's instructions. VSG cDNAs were produced from total RNA by RT-PCR as previously described [12]. The cDNAs were cloned into pGEM-T prior to being sequenced.

The genome sequences of *T. b. brucei *and *T. b. gambiense *were searched using the programme BLAST and the database resource [38]. The percentage identities were obtained from various BLAST programmes without manual optimization of alignments.

The multiple sequence alignments of VSG N-terminal domain sequences were produced using ClustalW. An empirical measure of sequence variation was directly imported from these alignments: positions where all three amino acids were identical (* in Clustal W) scored 0; a conservative substitution (: in Clustal W) scored 1; decreasingly conservative substitutions scored 2 (. in Clustal W) and divergent substitutions scored 3.

Solvent accessibility was calculated as the relative accessibility of each residue × as a percentage when compared to that residue type in an extended alanine-X-alanine tripeptide (Hubbard S.J. [39]).

## Abbreviations

RT-PCR reverse transcriptase and polymerase chain reaction

VSG variant surface glycoprotein

## Authors' contributions

CH, KP and RS carried out the experimental work. NJ, HM and MC analysed the data. SW and MC designed the study. All authors read and approved the manuscript.

## Supplementary Material

Additional file 1 1. Map showing location where isolates were collected. 2. A comparison of VSGs AnTat1.1b and AnTat 1.10. 3. The following data are presented for each VSG : a) Amino acid sequence with cysteine residues in red. b) Domain combination. c) N-terminal domain sequences of homologues identified using the whole VSG sequence to screen the *T. b. brucei *TREU927 and *T. b. gambiense *Daloa genomic sequences. The N-terminal domain was standardised by ending 5 residues before first cysteine in the C-terminal domain. d) Percentage identities measured using NCBI blast 2 sequences. e) Three way alignment for the six VSGs used to determine the location of sequence divergence. Click here for file
