# Supplementary material for: Variant Surface Glycoprotein gene repertoires in Trypanosoma brucei have diverged to become strain-specific
Source: BMC Genomics. 2007 Jul 13;8:234. doi: 10.1186/1471-2164-8-234 (PMC1934917; doi:10.1186/1471-2164-8-234)
Supplement: Additional file 1 — 1. Map showing location where isolates were collected. 2. A comparison of VSGs AnTat1.1b and AnTat 1.10. 3. The following data are presented for each VSG : a) Amino acid sequence with cysteine residues in red. b) Domain combination. c) N-terminal domain sequences of homologues identified using the whole VSG sequence to screen the T. b. brucei TREU927 and T. b. gambiense Daloa genomic sequences. The N-terminal domain was standardised by ending 5 residues before first cysteine in the C-terminal domain. d) Percentage identities measured using NCBI blast 2 sequences. e) Three way alignment for the six VSGs used to determine the location of sequence divergence. [file 1471-2164-8-234-S1.pdf]

Additional file 1

1. Map showing location where isolates were collected.

2. A comparison of VSGs AnTat1.1b and AnTat 1.10

3. The following data are presented for each VSG :

a) Amino acid sequence with cysteine residues in red

b) Domain combination

c) N-terminal domain sequences of homologues identified using the whole VSG sequence to screen the *T. b. brucei* TREU927 and *T. b. gambiense* Daloa genomic sequences. The N-terminal domain was standardised by ending 5 residues before first cysteine in the C-terminal domain

d) Percentage identities measured using NCBI blast 2 sequences (http://www.ncbi.nlm.nih.gov/blast/bl2seq/wblast2.cgi)

e) Thrre way alignment for the six VSGs used to determine the location of sequence divergence.

The screen was performed in 2006

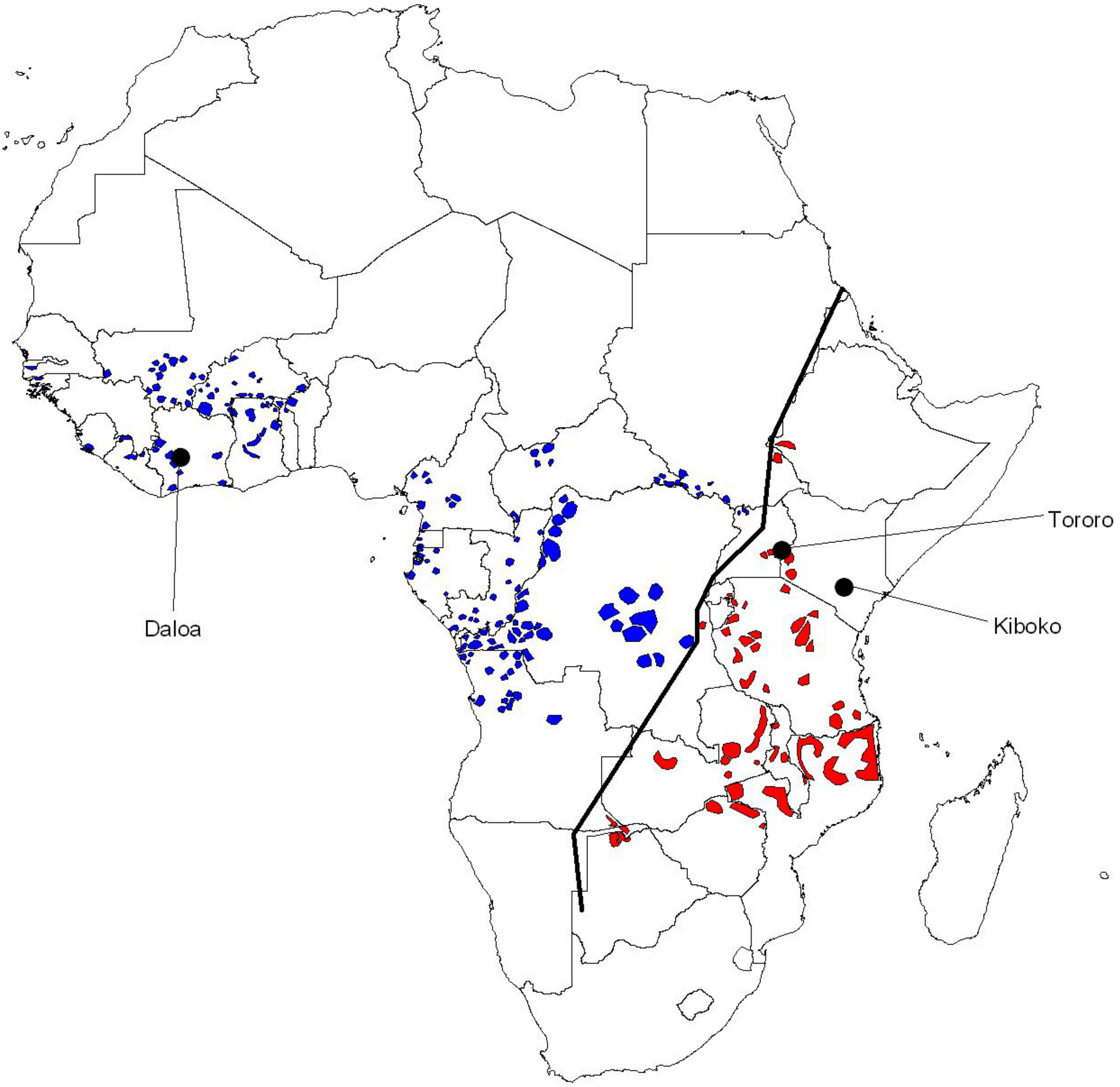

Map of Africa showing the geographic origins of the isolates analysed in this paper. The expressed VSGs were characterised from field isolates from Tororo in Uganda; the *Trypansoma brucei brucei* TREU927 genome reference strain came from Kiboko in Kenya and the *T. b. gambiense* genome strain came from Daloa in Cote d'Ivoire. Areas of endemic *T. b. rhodesiense* human trypanosomiasis are shown in red and *T. b. gambiense* human trypanosomiasis in blue. The range of *T. b. brucei* is far more extensive.

Location of differences between AnTat1.1 and AnTat1.10 in primary and tertiary structure of N-terminal domains

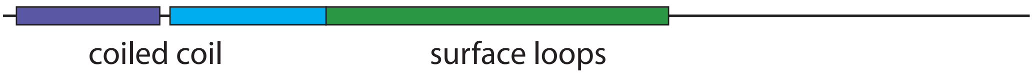


```
>Ant1.10
QTAGRPLADAVGKALCTYSKTAKRQAANLAQALDRGITAAKKSQ
QAHELATIALAKLPHYREAAATILIYAKNKRAEAEANIENWKGQ
KTKLVGEAMYSSGRIDELMLMLEGHRDGQSAGQTKTCLGAAGNG
NTVDEFVKTECDTEQDHNINADESDIEQAARTLSQENRDPEAGG
GTNCKITGNLASDYDSHPNDLSLLGGLLTIHNGGGFKATTTIKT
AAAANKLISALASKVNDIAANLKAHTESAPTTKQELKTLLAAKG
ARSKLAAANDEYTSWEAGKKPVNFDEHIKKVFGAEDGKDSAYAL
ALEGISIEVPQKPGTTESKQLYSMQPKDLMAALIGTIAEIQKAA
ATKAPCPKHKLTSA

>Ant1.1b
QTAGRPLADVVGKTLCTYSKTAKRQAANLAQTLQRASSAAKQSR
QAQQLAALALAKLPDYKEAAATLLIYATHKIQDAQASIENWTGE
NTKLVGQAMYSSGRIDELMLLLEGHREDGANGQDKTCLGAAAGG
NTVNEFVKTECDTESGHNIEADNSNIGATDYDSHANELPLLGGL
LTIHNAGGFKTGQSLQTAAPTNKLISALKNKGAVAAKLATVTA
ARPTNKQEFKTLLASKAERAKLQAANEEYNNWKPGAKPADFDAH
IKKVFGAEDGKDSAYALALEGISIEVPQKPGTTESKQLYSMQPK
DLMAALIGTIAEIQKAAATKAPCPKHKLTSA


Score =  437 bits (1125),  Expect = 4e-121
 Identities = 254/366 (69%), Positives = 286/366 (78%), Gaps = 27/366 (7%)

Ant1.10   1    QTAGRPLADAVGKALCTYSKTAKRQAANLAQALDRGITAAKKSQQAHELATIALAKLPHY  60
               QTAGRPLAD VGK LCTYSKTAKRQAANLAQ L R  +AAK+S+QA +LA +ALAKLP Y
Ant1.1b   1    QTAGRPLADVVGKTLCTYSKTAKRQAANLAQTLQRASSAAKQSRQAQQLAALALAKLPDY  60

Ant1.10   61   REAAATILIYAKNKRAEAEANIENWKGQKTKLVGEAMYSSGRIDELMLMLEGHRDGQSAG  120
               +EAAAT+LIYA +K  +A+A+IENW G+ TKLVG+AMYSSGRIDELML+LEGHR+  + G
Ant1.1b   61   KEAAATLLIYATHKIQDAQASIENWTGENTKLVGQAMYSSGRIDELMLLLEGHREDGANG  120

Ant1.10   121  QTKTCLGAAGNGNTVDEFVKTECDTEQDHNINADESDIEQAARTLSQENRDPEAGGGTNC  180
               Q KTCLGAA  GNTV+EFVKTECDTE  HNI AD S+I
Ant1.1b   121  QDKTCLGAAAGGNTVNEFVKTECDTESGHNIEADNSNIG---------------------  159

Ant1.10   181  KITGNLASDYDSHPNDLSLLGGLLTIHNGGGFKATTTIKTAAAANKLISALASKVNDIAA  240
                     A+DYDSH N+L LLGGLLTIHN GGFK   +++TAA  NKLISAL +K   +AA
Ant1.1b   160  ------ATDYDSHANELPLLGGLLTIHNAGGFKTGQSLQTAAPTNKLISALKNKGAGVAA  213

Ant1.10   241  NLKAHTESAPTTKQELKTLLAAKGARSKLAAANDEYTSWEAGKKPVNFDEHIKKVFGAED  300
                L   T + PT KQE KTLLA+K  R+KL AAN+EY +W+ G KP +FD HIKKVFGAED
Ant1.1b   214  KLATVTAARPTNKQEFKTLLASKAERAKLQAANEEYNNWKPGAKPADFDAHIKKVFGAED  273

Ant1.10   301  GKDSAYALALEGISIEVPQKPGTTESKQLYSMQPKDLMAALIGTIAEIQKAAATKAPCPK  360
               GKDSAYALALEGISIEVPQKPGTTESKQLYSMQPKDLMAALIGTIAEIQKAAATKAPCPK
Ant1.1b   274  GKDSAYALALEGISIEVPQKPGTTESKQLYSMQPKDLMAALIGTIAEIQKAAATKAPCPK  333

Ant1.10   361  HKLTSA  366
               HKLTSA
Ant1.1b   334  HKLTSA  339
```

Ako1

MPTVASMQHHKPIKASNIFFRYLTLALTLLVALSLTRKGESAAGD
GLNAAEFRALCHLAAIADLSADATTLPTIDPNEKQEIEMLNMSASQATWQAAFPAPDQPE
PDVQQACNTGDKRIHCLKDYKKFKQLKATLKTESAAEQTRGKHKLVPAIQETPEGRRVQR
QLHDLATEAADIISTYNSQHSQLKQKLGTALTTVLKQALYGTGTAEKTGKYGDKWTATAT
RTTDCAQNMAGTSLRGDLACLCINDSATNKQMCGNTVGPDNSNNWQASATTTNINGLVSK
CKTLVKPKLSAQTIRAALAIFDAKLRSHTGGGKDAIVLGTPHTNGDCGGQANVACVDYTQ
QLTPTNEGEQNKINWYQHLDQAASTLQELETAEIDRQHALRQLGDLKKRAYSLYNTLKIA
NSPHTTKGTEIHAKEQNTKESKCKLKNTTAEECPETDCDYDKTKNECKSKPGSETTATPT
GAGAAGTTTEKCKDKKKDDCKDGCKWEGETCKDSSILATKKFALSVVSAAFVALLF

Domain combination: B1

GeneDB Blast results N-terminal domains

>Ako1
MPTVASMQHHKPIKASNIFFRYLTLALTLLVALSLTRKGESAAGD
GLNAAEFRALCHLAAIADLSADATTLPTIDPNEKQEIEMLNMSASQATWQAAFPAPDQPE
PDVQQACNTGDKRIHCLKDYKKFKQLKATLKTESAAEQTRGKHKLVPAIQETPEGRRVQR
QLHDLATEAADIISTYNSQHSQLKQKLGTALTTVLKQALYGTGTAEKTGKYGDKWTATAT
RTTDCAQNMAGTSLRGDLACLCINDSATNKQMCGNTVGPDNSNNWQASATTTNINGLVSK
CKTLVKPKLSAQTIRAALAIFDAKLRSHTGGGKDAIVLGTPHTNGDCGGQANVACVDYTQ
QLTPTNEGEQNKINWYQHLDQAASTLQELETAEIDRQHALRQLGDLKKRAYSLYNTLKIA
NSPHTTKGTEIHAKEQN

>gamb276c12
MPTVASMQHHKPIKASNIFFRYLTLALTLLVAISLTRKGESAAGDGLNAAEFRALCHLAA
IADLSADGTTLPAINPNEKQEIEILNMSASQATWQAAFPDPDQPEPDVQQACNTGDKRIH
CLKDYKKFKQLKATLKTESAAEQTRGKHKLVPAIQETPEGRRIQKQLNDLAAEAADIISS
YESQHAQLKQKLGGALTSVLKQALYGTRTTQTGSTYADKWTATGSRTADCEAQKAGLSLR
GDLACLCISDSTTRKQMCGNGIGTDSSSEWQTSASKINIDSLISKCKEITKPKLRAATIR
TALAIFDAKLKSHTQGSKDAIVLGTPHTNGNCGQQASVACVDYTQQLTPTNEGEQNKINW
YQHLDQAASTLQELETAEIERQHALRQLGALKKRAYSLYNTLKIANSPHTTKGTEIHAKE
QN

>contig11608 adjusted manually to maintain VSG reading frame
AEFRALCRLPPIPDLLANATALPTIDPNEKQEIEILNMSASQATWQAAFPAPDQADPEPE
PACKSEDQVVRCLKDYQTFKQLKESLKSESAAEKTGDKHKLVPAIQET
PEGRRVQRQLHDLAAESAEVISSYDSQHSQLKQKLGAALTSILKEALYGAGTTATGENYD
DKWTATTTRAADCAAPQAGKSIRGDLACLCINDQTTSKQMCGNAVGTDGGNDWQASATAT
NIDKLVGKCKTLVKPKLTTRAIHKALSDFDAKLKSHTGGAKDAIVLGTPHTDGTCGSTAN
KACVDYTEQLTPTTEGAQNKISWYQHLEHAART
LQELQTAQIERQDALRRITTLKKRAYSLYNTLKIANSPYTTKGTEIHAKEQN
Short at N-terminus due to end of contig – couldn't extend

NCBI blast 2 sequence scores for N-terminal domains excluding N-terminal signal sequences

Tororo v Tbg: 84%
Tororo v Tbb 927: 73% over available sequence
Tbg v Tbb927: 71% over available sequence

```
CLUSTAL W (1.83) multiple sequence alignment


Ako1            MPTVASMQHHKPIKASNIFFRYLTLALTLLVALSLTRKGESAAGDGLNAAEFRALCHLAA 60
TbgDal          MPTVASMQHHKPIKASNIFFRYLTLALTLLVAISLTRKGESAAGDGLNAAEFRALCHLAA 60
Tbb927          -------------------------------------------------AEFRALCRLPP 11
                                                                 *******:*..

Ako1            IADLSADATTLPTIDPNEKQEIEMLNMSASQATWQAAFPAPDQPEPDVQQACNTGDKRIH 120
TbgDal          IADLSADGTTLPAINPNEKQEIEILNMSASQATWQAAFPDPDQPEPDVQQACNTGDKRIH 120
Tbb927          IPDLLANATALPTIDPNEKQEIEILNMSASQATWQAAFPAPDQADPEPEPACKSEDQVVR 71
                *.** *:.*:**:*:*******:*************** ***.:*: : **:: *: ::

Ako1            CLKDYKKFKQLKATLKTESAAEQTRGKHKLVPAIQETPEGRRVQRQLHDLATEAADIIST 180
TbgDal          CLKDYKKFKQLKATLKTESAAEQTRGKHKLVPAIQETPEGRRIQKQLNDLAAEAADIISS 180
Tbb927          CLKDYQTFKQLKESLKSESAAEKTGDKHKLVPAIQETPEGRRVQRQLHDLAAESAEVISS 131
                *****:.***** :**:*****:* .****************:*:**:***:*:*::**:

Ako1            YNSQHSQLKQKLGTALTTVLKQALYGTGTAEKTGKYGDKWTATATRTTDCAQNMAGTSLR 240
TbgDal          YESQHAQLKQKLGGALTSVLKQALYGTRTTQTGSTYADKWTATGSRTADCEAQKAGLSLR 240
Tbb927          YDSQHSQLKQKLGAALTSILKEALYGAGTTATGENYDDKWTATTTRAADCAAPQAGKSIR 191
                *:***:******* ***::**:****: *: .  .* ****** :*::**    ** *:*

Ako1            GDLACLCINDSATNKQMCGNTVGPDNSNNWQASATTTNINGLVSKCKTLVKPKLSAQTIR 300
TbgDal          GDLACLCISDSTTRKQMCGNGIGTDSSSEWQTSASKINIDSLISKCKEITKPKLRAATIR 300
Tbb927          GDLACLCINDQTTSKQMCGNAVGTDGGNDWQASATATNIDKLVGKCKTLVKPKLTTRAIH 251
                ********.*.:* ****** :*.*...:**:**:  **: *:.*** :.**** : :*:

Ako1            AALAIFDAKLRSHTGGGKDAIVLGTPHTNGDCGGQANVACVDYTQQLTPTNEGEQNKINW 360
TbgDal          TALAIFDAKLKSHTQGSKDAIVLGTPHTNGNCGQQASVACVDYTQQLTPTNEGEQNKINW 360
Tbb927          KALSDFDAKLKSHTGGAKDAIVLGTPHTDGTCGSTANKACVDYTEQLTPTTEGAQNKISW 311
                 **: *****:*** *.***********:* **  *. ******:*****.** ****.*

Ako1            YQHLDQAASTLQELETAEIDRQHALRQLGDLKKRAYSLYNTLKIANSPHTTKGTEIHAKE 420
TbgDal          YQHLDQAASTLQELETAEIERQHALRQLGALKKRAYSLYNTLKIANSPHTTKGTEIHAKE 420
Tbb927          YQHLEHAARTLQELQTAQIERQDALRRITTLKKRAYSLYNTLKIANSPYTTKGTEIHAKE 371
                ****::** *****:**:*:**.***::  ******************:***********

Ako1            QN 422
TbgDal          QN 422
Tbb927          QN 373
                **
```

Ako 2

MSAVATGFILAIAALKQAGAVSDGENAAIFKPLCAALQLAD
VKPTFEPPIQPKMPEPLELYRLNMSIAPKDWRAKFLKQSTAPPTAVDLPQGEKDAERQTR
WKTWTDTAVFLATGSNEKDLKSNYGLTTATDAQIEAIRATIHDIVETAHAIYTATATANE
TAPADDALLQKEIAQAVYGEETYGPAELTATKVLSGADAGYTAACGGGGTHKPQNTVAGT
IICVCGCAEGQSHKPCHKKQATETAWQATNIPNRASWGNLRNVCPKATKTKITAHGLREL
VAAAKKIANTQGNDVFIGSEGENTCNGNANGACVKITGGVASGNLKDDAIPWINKLEAVA
EQLTERGNYNSEQQRKTAAINELISRAKAVAKRAHYLFQFQKTAQAAAAGEATEGTVSKA
DCKQYTTNSTCPKNDCKWDSTTEDKGDHCKPKPGSEPTTAGTGDGDAKTDSKCSDKKKEE
DCKDGCKWDGKECKDSSILVNKQFAFSVVSAAFMALLF

Domain combination: B1

GeneDB Blast results N-terminal domains

>ako2
MSAVATGFILAIAALKQAGAVSDGENAAIFKPLCAALQLAD
VKPTFEPPIQPKMPEPLELYRLNMSIAPKDWRAKFLKQSTAPPTAVDLPQGEKDAERQTR
WKTWTDTAVFLATGSNEKDLKSNYGLTTATDAQIEAIRATIHDIVETAHAIYTATATANE
TAPADDALLQKEIAQAVYGEETYGPAELTATKVLSGADAGYTAACGGGGTHKPQNTVAGT
IICVCGCAEGQSHKPCHKKQATETAWQATNIPNRASWGNLRNVCPKATKTKITAHGLREL
VAAAKKIANTQGNDVFIGSEGENTCNGNANGACVKITGGVASGNLKDDAIPWINKLEAVA
EQLTERGNYNSEQQRKTAAINELISRAKAVAKRAHYLFQFQKTAQAAAAGEATEG

>Tb11.24.0011
MSAVAPGVILAIAALKQARGVTDGDNAAIFKPLCAALQLADVKPTFEPPIQANMPE
PLDLYRLNMSIAPKDWRAKFLNQGNKAASTPAEVPTDENDEELKARWKTWADTAVFLATD
NNEKDLKANYGLATATAGQIEAIRPTIHDITEAARAIYTADSDPGPAPDADELLQKEIAQ
AVYGQEQWGPEELTANKVLSGPDTGYTTACGGGGTQKPQNTVAGTIICVCGTGNTQSKKP
CHKKQTTQTAWQATNIPNRASWGNLRNVCPKATKTKITAHGLRELVAAAKTIANTQGNNV
FIGSEGENTCNGNAAGACVKITGGAPSGNLKDDAIPWINKLEAVADKLAARDNYNSEQRR
KKAAIADLISRAKAVTKRAHYLFQFQKTAKTAAAGEATEG

>Tbg843f08.p1k
MSAVATGVILAIAALTQARGVTDGDNAAIFKPLCATLQLADAKSTFEPPIQPKMPEPLDL
YRLNMSIAPKNWRAKFLKQYNKATPTPAAADLPQGEKDAETTARWKTWADTAVFLATDNN
EKDLQANYGLTTATAGQIEAIRPTIHDITEAARAIYTADSDPETTPEDDELLQK*IAEA
VYGQEQWGPEELTANKVLSGPDTGYTTACGGSGTQKPQNTVAGTIICVCGCANTQTHKPC
HKKQTAETAWQGSNIPDRSSWGNLRTICPKATKTKITAHGLRELVAAAKTIANTQGNDVF
IGSEGENTCNGNANGACVKITGGAASGNLKDDAIPWINKLEAVADKLAARDNYNSEQRRK
AAAIADLISRAKAVTKRAHYLFQFQKTAKTAAAGEATEG

NCBI blast 2 sequence scores for N-terminal domains excluding N-terminal signal sequences

Tororo v Tbg: 79%
Tororo v Tbb 927: 79%
Tbg v Tbb927: 87%

```
CLUSTAL W (1.83) multiple sequence alignment


Tbb927          MSAVAPGVILAIAALKQARGVTDGDNAAIFKPLCAALQLADVKPTFEPPIQANMPEPLDL 60
TbgDal          MSAVATGVILAIAALTQARGVTDGDNAAIFKPLCATLQLADAKSTFEPPIQPKMPEPLDL 60
ako2            MSAVATGFILAIAALKQAGAVSDGENAAIFKPLCAALQLADVKPTFEPPIQPKMPEPLEL 60
                *****.*.*******.** .*:**:**********:*****.*.*******.:*****:*

Tbb927          YRLNMSIAPKDWRAKFLNQGNKAASTP--AEVPTDENDEELKARWKTWADTAVFLATDNN 118
TbgDal          YRLNMSIAPKNWRAKFLKQYNKATPTPAAADLPQGEKDAETTARWKTWADTAVFLATDNN 120
ako2            YRLNMSIAPKDWRAKFLKQS---TAPPTAVDLPQGEKDAERQTRWKTWTDTAVFLATGSN 117
                **********:******:*    :..*  .::* .*:* *  :*****:********..*

Tbb927          EKDLKANYGLATATAGQIEAIRPTIHDITEAARAIYTADSDP-GPAPDADELLQKEIAQA 177
TbgDal          EKDLQANYGLTTATAGQIEAIRPTIHDITEAARAIYTADSDP-ETTPEDDELLQK-IAEA 178
ako2            EKDLKSNYGLTTATDAQIEAIRATIHDIVETAHAIYTATATANETAPADDALLQKEIAQA 177
                ****::****:*** .******.*****.*:*:***** : .  .:*  * **** **:*

Tbb927          VYGQEQWGPEELTANKVLSGPDTGYTTACGGGGTQKPQNTVAGTIICVCGTGNTQSKKPC 237
TbgDal          VYGQEQWGPEELTANKVLSGPDTGYTTACGGSGTQKPQNTVAGTIICVCGCANTQTHKPC 238
ako2            VYGEETYGPAELTATKVLSGADAGYTAACGGGGTHKPQNTVAGTIICVCGCAEGQSHKPC 237
                ***:* :** ****.*****.*:***:****.**:*************** .: *::***

Tbb927          HKKQTTQTAWQATNIPNRASWGNLRNVCPKATKTKITAHGLRELVAAAKTIANTQGNNVF 297
TbgDal          HKKQTAETAWQGSNIPDRSSWGNLRTICPKATKTKITAHGLRELVAAAKTIANTQGNDVF 298
ako2            HKKQATETAWQATNIPNRASWGNLRNVCPKATKTKITAHGLRELVAAAKKIANTQGNDVF 297
                ****:::****.:***:*:******.:**********************.*******:**

Tbb927          IGSEGENTCNGNAAGACVKITGGAPSGNLKDDAIPWINKLEAVADKLAARDNYNSEQRRK 357
TbgDal          IGSEGENTCNGNANGACVKITGGAASGNLKDDAIPWINKLEAVADKLAARDNYNSEQRRK 358
ako2            IGSEGENTCNGNANGACVKITGGVASGNLKDDAIPWINKLEAVAEQLTERGNYNSEQQRK 357
                ************* *********..*******************::*: *.******:**

Tbb927          KAAIADLISRAKAVTKRAHYLFQFQKTAKTAAAGEATEG 396
TbgDal          AAAIADLISRAKAVTKRAHYLFQFQKTAKTAAAGEATEG 397
ako2            TAAINELISRAKAVAKRAHYLFQFQKTAQAAAAGEATEG 396
                 *** :********:*************::*********
```

Bug1

```
>Bug1
MQALRRTLTISFVAALAWPQQASGSGNALKTAAWKEMCQTAKEL
RAVPTAAANKLASTRQTIAELEATALRLAAVAASQADVGDAATLGALAKELSTEAASL
SSGYTAKTNKALTAAAASAAVYGGISEFLNLLTAGITGATNGCLAKQDGATLIGSRDE
LTDCPIDKELTVEETSSTIKTTFTADGITTLKTDNVKDTGSNAALCVLFQTPTDAPSK
LFHKAAEFLAAGGTLSVNTATTTIKTEHGKKISKETNKVGTALIKQAHTQTHELLTEP
ENDPTSASKSKIKALASDTNFTATVVRQLKLIGMAGSDKTLEDYAKSKIKTLLDDGKA
NFDTKWNALIDTKIYDGKSEKVKTEKASSITSTKELIQSVLYYQIDLKTKLKRVKEEL
EQKKNDCEKETEEVCNAIEGEQECNNTKDCHYDSKKDGKKCTLKKEVKEKLEKTNQET
GVKDGNKDRCTKHGTDKTKCEAENTAGQTPVCGFRKGKDNEPEQDKEMCRNGSFLVNN
KFALSMVSAAFVALLF
```

Domain combination: A1

GeneDB Blast results N-terminal domains

Tbg:
Contig assembled manually based on:

\>gamb1660g03.q1krevcomp
>gamb187e12.p1k
>gamb1588a05.p1k

```
>Bug1
MQALRRTLTISFVAALAWPQQASGSGNALKTAAWKEMCQTAKEL
RAVPTAAANKLASTRQTIAELEATALRLAAVAASQADVGDAATLGALAKELSTEAASL
SSGYTAKTNKALTAAAASAAVYGGISEFLNLLTAGITGATNGCLAKQDGATLIGSRDE
LTDCPIDKELTVEETSSTIKTTFTADGITTLKTDNVKDTGSNAALCVLFQTPTDAPSK
LFHKAAEFLAAGGTLSVNTATTTIKTEHGKKISKETNKVGTALIKQAHTQTHELLTEP
ENDPTSASKSKIKALASDTNFTATVVRQLKLIGMAGSDKTLEDYAKSKIKTLLDDGKA
NFDTKWNALIDTKIYDGKSEKVKTEKASSITSTKELIQSVLYYQIDLKTKLKRVKEEL
E

>Tbb927chr5
MHIIARIIAFTYVAVLMCTQRSATTGAALKKAAWNKMCATEKELRTVPATAEQKLTEAQG
TIEALENPALKMAAVAGAHADAGEALILAALAAELRSEAQTKLLNLKELRKTALEAAAVA
AAVYGGVSEFFTLMTAGQSGTINGCLADTDGSNVISGKSGLGDCPIDQAISLTDTSETLT
TTFTENGISSLKAADVKDDSTNANKCVLFQNAQAGNDRLFQHGAAFLAAGGTLSVSTAAT
TLKTGHGQNIKANSTATGAALIKQAHSATLELLKHATTEAEHVGKNKAKELASDSHFAAT
VVKKLKLIGMKGSDSTLEAYAKSKIKSLLADGGENFGSKWQTLIDTPIYDGKSEEVKTEK
ASSITSTKELIQSVLYYQIDLKTKFKRAKEELE

>Tbg
MSRAKATTSFIFAAVLVCSQLGAATGNALKAAAWQDMCTTAE
QLTKIPALATKKLDQAQAVMADYRDAAMRLTAVAATRADSGDAIVLAILGSKLLEAAEET
AGNLKALRPLSIKAAAAAGTVYGGISDFLNLLSAAHTGITCGCLNAKGDGDIMADTTELA
SCPIDNEIDLEKDTTGLETVVGDKGFKTLKTTDVKATSTSNTKCVLFQDASAVGNNKLFQ
KAATFLIAGGTLKVNSAGPTMTTAHGAALAAEQSTAGGKLITQAHAAIKELLSQAETAPA
SAAKTKVKALAADSNFAKVVVNKLKLIGMEGSGNTLEHNAKVKIKNLLADGCKNFDSKWQ
ALIDTPIYDGKSEKIKTEKASSISSTTEIIQSVLYYQTKQQTDFLRLQKELE
```

NCBI blast 2 sequence scores for N-terminal domains excluding N-terminal signal sequences

Tororo v Tbg: 50%
Tororo v Tbb 927: 58%
Tbg v Tbb927: 50%

Bug2

MLATAETRQTARLLTLQTAVLAALVIPRSADAAAAHSSSGISG
FRAICELINLGAASCQDDQVGAESNDIKEAAALINLTIANPAIITELEAKATPEEAIGTE
NSKAAQQCTGDNEWICKAAHSRLKQKKGLKTKQTLTELSTKPTLKESIEAVLNQLKTEIA
SIDNRSGAKQCSEAKKALATAIGVQDTSKDNLALPEATTDRATTCGKPSGTDKGTIAGKN
LLADAVCLCSSDAASSNAGDNCNLQTGTGNLDFASAISVSSSDLKKLLGGCSAFKADTKL
TAHALEAALSAFDNERFRNQGTNNKITNVIGHVDGTGAECDGSDAGNKGACVYYGKSPGS
KALLPPAWREHITAAIGHLQHAEQERLRRHESIKRATTLNTTLAALILIAGDASSSQALR
EAAQTKQTNIGAQPGGKGAKDCGQHKDNTTCTAHKNCKWEGESDTKGECKPKDGEGQTNP
AAGAGTDGKTNTTGSNSFAINKAPLLLAFLLF

Domain combination: B2

GeneDB Blast results N-terminal domains

Tbb 927 – best match CONTIG11866 32%

>Bug2
MLATAETRQTARLLTLQTAVLAALVIPRSADA
AAAHSSSGISG
FRAICELINLGAASCQDDQVGAESNDIKEAAALINLTIANPAIITELEAKATPEEAIGTE
NSKAAQQCTGDNEWICKAAHSRLKQKKGLKTKQTLTELSTKPTLKESIEAVLNQLKTEIA
SIDNRSGAKQCSEAKKALATAIGVQDTSKDNLALPEATTDRATTCGKPSGTDKGTIAGKN
LLADAVCLCSSDAASSNAGDNCNLQTGTGNLDFASAISVSSSDLKKLLGGCSAFKADTKL
TAHALEAALSAFDNERFRNQGTNNKITNVIGHVDGTGAECDGSDAGNKGACVYYGKSPGS
KALLPPAWREHITAAIGHLQHAEQERLRRHESIKRATTLNTTLAALILIAGDASSSQALR
EAAQTKQTNIGAQPGG

>gamb856d03.q1k_1
MLATAETRQTARLLTLQTAVLAALVIPRSGSAA
AAHTSSGISGFRAICELINLGDASCQE
DQVGAESNDIKEAAALINLTIANPAIITELEAKDTAEEAIGTENSKAAQQCSGDNEWICK
AAHRRLKQKKGLKTKQTLTELSTKPTLKESIEAVLNQLKTEIASLDNRDGAKHCSEAKKA
LATAIGVQDTTKDNLNLPEATTDRATTCGKPSGTHKGTIAGKNLLGDAVCLCSSDATSSN
AGENCNLQTGTANLDFRSAISVTNSDLKKLLGGCSAFKADSKLTAHALEAALSAFDSERF
RNQGTNNKITNVIGHVDGSGNECDGSNDGNKGACVYYGKSPGSKALLPPAWREHITAAIG
HLQHAEQERLRRHESIKRATTLNTTLAALILIAGDASSNQALREAAQTKQTNIGAQPGG

NCBI blast 2 sequence scores for N-terminal domains
excluding n-terminal signal sequences

Tororo v Tbg: 93%
Tororo v Tbb 927: N/A
Tbg v Tbb927: N/A

Buteba 1

>Buteba 1
MSMLSLTIRKRQPQNAITNAIATTTVFISIILLAINSGATAPTTAVNAREFDLLCTLVRA
EENLEERQTASQAAKEVVALAAQIELILANLKHIERLAAAEPQDAPKESSSGDTPEACKA
PKSTVCTKAAQIYKRFRPDEKLALAFLAETTGELRATFNVTLKQIATTATSHARYFGQNT
ETRQALAKIKKALYGSPEAKGDAIIDSADSTRSAACGKTDSNEANSAKKRAAAALICLCG
GDNTNTGNDACFTQTKADINYASKSGAVEKAWTEISQKCQANTRTTKVTTAQLKTAATEL
ATLIHQKRGDKAVAGLLGAAQINAGAVDCDGSDANGKGSCVILSTSAKKYKVETPDWLTA
MQEAINNLDDEQIEINNGQKAEAHILALNSSLTTLLAQAVAPAKQAPTQAAVPPEKQTNP
QKDCTKNTKKKDCKEGDGCKWTNEDEETGSHCKAKNDGEGVKTENEGTTTTSATGNNSFV
IKTSPLLLAVLLF

Domain combination: B2

GeneDB Blast results N-terminal domains

Tbg:
Two non-overlapping hits with
>gamb333c09.p1k
>gamb215b11.p1k
cannot join or extend to form contigs

>But1
MSMLSLTIRKRQPQNAITNAIATTTVFISIILLAINSGATAPTT
AVNAREFDLLCTLVRA
EENLEERQTASQAAKEVVALAAQIELILANLKHIERLAAAEPQDAPKESSSGDTPEACKA
PKSTVCTKAAQIYKRFRPDEKLALAFLAETTGELRATFNVTLKQIATTATSHARYFGQNT
ETRQALAKIKKALYGSPEAKGDAIIDSADSTRSAACGKTDSNEANSAKKRAAAALICLCG
GDNTNTGNDACFTQTKADINYASKSGAVEKAWTEISQKCQANTRTTKVTTAQLKTAATEL
ATLIHQKRGDKAVAGLLGAAQINAGAVDCDGSDANGKGSCVILSTSAKKYKVETPDWLTA
MQEAINNLDDEQIEINNGQKAEAHILALNSSLTTLLAQAVAPAKQAPTQAAVPPEKQT

>Tb10.v4.0152
MSMLSLTNRKQQLQNAITNAIATTTVLISIILLAINSGAAAPTT
AVNAREFDLLCTLVRA
EENLEERQTAGQAAKEVVALAAKIELILANLKHIERLAAAEPQAAPKESTSDETPEACKA
AKSTVCNKAAQIYKKFRPDEKRALAFLAETTDKLSATVNVTLKQIADNKANHAAYFGEST
GKRPAFANVKKALYGDPAVTGDGIIDTGDNTRPKACGNTASAAAKSGKARAEAALICLRG
SDSSNGGNAACFTGAALDTNYGSPSNDFATAWTQIKQQCSQKQRQAKVTGEQLKTAASAL
NHLVHSKHGDKAVAGLLGYAEANTGAISCDGSTGNGKGSCVILSTSANSYKVETPDWLTA
MQETINNLDGEKHEIEKGWKAEAHTLALNSSLTTLLALQAGSSKEAAPEKQASPEKQS

>Tbg
MSMLSLTPRKQELRSAITNAITTTAVFINTIPLAINSDAAGPTK
AVNAREFELLCTLVQA
EDNLEARLSAGAESKSVVKLAEQLEIILPNLKHIERLAAAEPQAAPKESSSDETPEACKA
AKSTVCDKAAQIYKKLHPDEKLELAFLAETTSKL
gap
TRPTACGNTASAVANSAKKRAAAALICLCG
SDNTNTANAACFTGAAPNTNYGSPSNDFATAWTQIKQQCSQKQKQAKVTGEQLKTAASPL
NHLVHSKHGDKAIVGLLGYAEANTGAISCDGSTGNGKGSCVILSTSANSYKVETPDWVTA
MQETINNLDGEKHEIEKGWKAEAHTLALNSSLTTLLALPAGSSKEAAPEKQASPEKQS

NCBI blast 2 sequence scores for N-terminal domains excluding N-terminal signal sequences

Tororo v Tbg: 67% (for available sequence)
Tororo v Tbb 927: 69%
Tbg v Tbb927: 87% (for available sequence)

Buteba 2

MAKISSKIEVSYIVLPLSLIFAVSGCHATQGDALSKENRDELCNLNKELAVL
PATYVKNLISQWDAEDLRASDELRLRIYLADNTPQKAAELAPLLAAHSAIVQRTATETAT
ETKEVAGLIAAAAALNGQITEFFHIAANAYQAGGTGKGCVTGEDASKIVQGTKGLGDCGL
ETLTVEPTAAAALTQLTATGFTNLQGAKTTTQRADASADCRLFNTDGSNGVLGSQASAGG
LTFAGGYIKVAANADTITGSSLAAMNEGHEATHKAYHRLWRAYVTITKSKAATQAGYSRI
NLDQLTGTTEARRVFREAIQKTPKYDDAKDSDTVKQSMTAHYGPDAKFNSKTWELVDGHQ
VKKEAYETNGKGTEKLANIKDINELRKILTYYQTRQTSISNNAAQETKQCSEITAQEQKP
PETDTTCEKKGTGAECKDGCKVEGTGDSKKCVKDPTYKPKQAEGVKAEGKGKTNTTGSNS
FVIKKSFLFLAFLLF

Domain combination: A2

GeneDB Blast results N-terminal domains

Tbb 927 best match Tb927.3.270 32% identity

>but2
MAKISSKIEVSYIVLPLSLIFAVSGCHA
TQGDALSKENRDELCNLNKELAVL
PATYVKNLISQWDAEDLRASDELRLRIYLADNTPQKAAELAPLLAAHSAIVQRTATETAT
ETKEVAGLIAAAAALNGQITEFFHIAANAYQAGGTGKGCVTGEDASKIVQGTKGLGDCGL
ETLTVEPTAAAALTQLTATGFTNLQGAKTTTQRADASADCRLFNTDGSNGVLGSQASAGG
LTFAGGYIKVAANADTITGSSLAAMNEGHEATHKAYHRLWRAYVTITKSKAATQAGYSRI
NLDQLTGTTEARRVFREAIQKTPKYDDAKDSDTVKQSMTAHYGPDAKFNSKTWELVDGHQ
VKKEAYETNGKGTEKLANIKDINELRKILTYYQTRQTSISNNAAQETKQCSEITAQEQKP
P

>Tbg1
AKISSKIEVSYIVLPLSLIFAVSGCHA
TQGDAL*KENRNELCNLNKEIAVLPATYVKNLI
SQSETEEGKATDELRLRIYLADSTPKKAAELAPLLAAHSAIVQRTATETATETEEVAGLI
AAAGALNGQITEYFHIAANAYQSGGSGKGCITADADSKIVQGPAGLTDCGLTTLSVDPVA
ATEITEVTKTVFPGLNARK
Gap
SQQSAGALVFAGGYITVAANTAEITATSLAAMNEGHATSHKAYHRLWKAYVTITKSKAAT
QATYSRINLADLTNTPEARRVFREAIQKTPKYDDAKDSDTVKQSMTAHXSPDVKFNSKTW
ELVDGHQVTKEGYETNGKGTEKLANIKDINELRKILTYYQTRQTSISNNAAQETKQCSEI
TAQGQKHQ

NCBI blast 2 sequence scores for N-terminal domains excluding n-terminal signal sequences

Tororo v Tbg: 84% over available sequence
Tororo v Tbb 927: N/A
Tbg v Tbb927: N/A

Buteba 3

```
MKLCIFCSVFIAVLQMVEIGASGAVTPGENAAEFAAVCSLV
QFAKAKIPPASEPQEIQKIINTLSVINFTLMDEQTRSTVEKNKEKTWEQIQSEHTGETKY
YGDHWDKWTRVAKLPPTDPEALALKDWERHRANENLKQQIKSLLHEALNLVKEAATFKED
LKAASLERLQELALYGSVGKSAELKSADDRKGYCGKGTTDGTAAGVGATDSLYATILCLC
AGAATDNTVGKGCCLDCQSGANAAAWTAHSNGKDRAEFLASKCPAALIPEHPTTPELNHR
LAAFYKLALGTTSTTQTIKYTVGKVSGTGEGGCDGEVTGTSGRCAKYTAQQILQDDASLK
WRQSLKAAATLYDKQAQAKITTYRLADKLRLINATAAALYHTPLTQTQIQPMSRTDAATK
PTTNCSEHKSSKTTCENTGKCEWEANPSDKSKGECKPKAGTETPAVGSGDGAAAGASATD
KKCSEKKTEGECKDGCKWEGTECKDSSILVNKKFALSVVCAAFAVLLS
```

Domain combination: B1

GeneDB Blast results N-terminal domains

gambiense contig assembled by hand starting with
>gamb1420g06.q1k
>gamb1557e09.q1k
>gamb1465h01.q1k
>gamb655g05.p1k

```
>but3
MKLCIFCSVFIAVLQMVEIGASGAVTPGEN
AAEFAAVCSLV
QFAKAKIPPASEPQEIQKIINTLSVINFTLMDEQTRSTVEKNKEKTWEQIQSEHTGETKY
YGDHWDKWTRVAKLPPTDPEALALKDWERHRANENLKQQIKSLLHEALNLVKEAATFKED
LKAASLERLQELALYGSVGKSAELKSADDRKGYCGKGTTDGTAAGVGATDSLYATILCLC
AGAATDNTVGKGCCLDCQSGANAAAWTAHSNGKDRAEFLASKCPAALIPEHPTTPELNHR
LAAFYKLALGTTSTTQTIKYTVGKVSGTGEGGCDGEVTGTSGRCAKYTAQQILQDDASLK
WRQSLKAAATLYDKQAQAKITTYRLADKLRLINATAAALYHTPLTQTQIQPMSRTDAAT

>927chr9
MKLCIFRSVFIAVLQIFEIGASGAAPPGEN
AAEFAAVCSLVQFAKAKIPPASEPQEIQKI
INTLSAINFTLMDDLTRSTVEKNK*KT*EQIQSEHTGETLYFGDH*DKWTRVAKLPETDP
EALALKDWERHRANNNLKKQIKSLL*EALALDKEAANAKEDLKVASITNLQEAALYGSPG
KSVELKTTDDRKGYFGKGSTDGAAAGVGATDSLYATILCLCAGATGDEAGGKGCCLDCQS
GANGAAWTPTSAGKARAEFLASKCPAALIPEHPTTTELNHRLRAVYKLALGTTSNTQTIK
YTVGKVSGTGEDGCGGEVTGTIGRCVKYTAEQILEDEASLK*RQSLKAAAKLYDKQAQAK
ITMYRLADKLRLINATAAAL*HTTLT*TQI*PMSRTDAAT

>tbg
MKLCIFCSVFIAVLQMFEIGATGAATPGEN
AAEFAAVCSLVQFAKAKIPPASEPQEIQKIINTLSAINFTLMYEQTRST
VEKNKEKTWEQIQSEHTGETKYYGDNWDKRTRVAKLPETDPEALALKDWERHRANNNLKN
QIKSLLWEALSLKTEAASAKEDLKAANLERLQELALYGSAGKSAELKSADDRKGYCGKGT
TDGTAAGVGATDLLYATILCLCAGAATDNTAGKGCCLDCQSGPNAEAWTAHSDGKDRAEF
LASKCPAALIPEHPTTPELNHRLAAFYKLALGTTSTTQTVKYTVGKVSGTCEGGCDGEVT
GTTGRCAKYTAQKILQDDASLKWRQSLKAAATLYDKQAQAKITTYRLADKLRLINATATA
LYHTTLTQTQIQPMSRTDAAK
```

NCBI blast 2 sequence scores for N-terminal domains
excluding n-terminal signal sequences

Tororo v Tbg: 92%
Tororo v Tbb 927: 84%
Tbg v Tbb927: 82%

CLUSTAL W (1.83) multiple sequence alignment

```
but3            MKLCIFCSVFIAVLQMVEIGASGAVTPGENAAEFAAVCSLVQFAKAKIPPASEPQEIQKI 60
tbgDal          MKLCIFCSVFIAVLQMFEIGATGAATPGENAAEFAAVCSLVQFAKAKIPPASEPQEIQKI 60
Tbb927          MKLCIFRSVFIAVLQIFEIGASGAAPPGENAAEFAAVCSLVQFAKAKIPPASEPQEIQKI 60
                ****** ********:.****:**..**********************************

but3            INTLSVINFTLMDEQTRSTVEKNKEKTWEQIQSEHTGETKYYGDHWDKWTRVAKLPPTDP 120
tbgDal          INTLSAINFTLMYEQTRSTVEKNKEKTWEQIQSEHTGETKYYGDNWDKRTRVAKLPETDP 120
Tbb927          INTLSAINFTLMDDLTRSTVEKNKKT--EQIQSEHTGETLYFGDH-DKWTRVAKLPETDP 117
                *****.****** : *********:.  *********** *:**: ** ******* ***

but3            EALALKDWERHRANENLKQQIKSLLHEALNLVKEAATFKEDLKAASLERLQELALYGSVG 180
tbgDal          EALALKDWERHRANNNLKNQIKSLLWEALSLKTEAASAKEDLKAANLERLQELALYGSAG 180
Tbb927          EALALKDWERHRANNNLKKQIKSLL-EALALDKEAANAKEDLKVASITNLQEAALYGSPG 176
                **************:***:****** *** * .***. *****.*.: .*** ***** *

but3            KSAELKSADDRKGYCGKGTTDGTAAGVGATDSLYATILCLCAGAATDNTVGKGCCLDCQS 240
tbgDal          KSAELKSADDRKGYCGKGTTDGTAAGVGATDLLYATILCLCAGAATDNTAGKGCCLDCQS 240
Tbb927          KSVELKTTDDRKGYFGKGSTDGAAAGVGATDSLYATILCLCAGATGDEAGGKGCCLDCQS 236
                **.***::****** ***:***:******** ************: *:: **********

but3            GANAAAWTAHSNGKDRAEFLASKCPAALIPEHPTTPELNHRLAAFYKLALGTTSTTQTIK 300
tbgDal          GPNAEAWTAHSDGKDRAEFLASKCPAALIPEHPTTPELNHRLAAFYKLALGTTSTTQTVK 300
Tbb927          GANGAAWTPTSAGKARAEFLASKCPAALIPEHPTTTELNHRLRAVYKLALGTTSNTQTIK 296
                *.*. ***. * ** ********************.****** *.*********.***:*

but3            YTVGKVSGTGEGGCDGEVTGTSGRCAKYTAQQILQDDASLKWRQSLKAAATLYDKQAQAK 360
tbgDal          YTVGKVSGTCEGGCDGEVTGTTGRCAKYTAQKILQDDASLKWRQSLKAAATLYDKQAQAK 360
Tbb927          YTVGKVSGTGEDGCGGEVTGTIGRCVKYTAEQILEDEASLK-RQSLKAAAKLYDKQAQAK 355
                ********* *.**.****** ***.****::**:*:**** ********.*********

but3            ITTYRLADKLRLINATAAALYHTPLTQTQIQPMSRTDAAT 400
tbgDal          ITTYRLADKLRLINATATALYHTTLTQTQIQPMSRTDAAK 400
Tbb927          ITMYRLADKLRLINATAAALHTTLTTQ---IPMSRTDAAT 392
                ** **************:**: *  **    ********.
```

Buteba 4

MPKATYALVVQTLLLVSKV
HQVQPTAGDAIKKKYWTALCDIAVDADKIAAKALHNLQQPATPGAESLRNLLRALVYNLA
NNTEPTSPGERMIWSHFAEEASAAIDFYKGDKPAKLITAVRDAARANGAILDWINTQHKV
STSSHGCLSGGDSGEAPAAGSAGMATIDAKCVPNWTAVTAEAAATTAVGLQGLQGTLGGQ
KGEGELANGGHGCNSNSANSAVKLLNGGTGTSVAGQSPTLVAGIFKLTGTSMAMDDLSNV
ATKQTTHPYVYHIVQAVKTAGETLATEDVSTIDRAKTSNSFKSVARVHLLGTKATDSRRD
TEIPDKIQAVFGSPDKYTAIYSKNVDEMPLTNELTHDPNLKKLGDITDINDLMRLYFFFL
DRLKSHVKDLTAQLEEAKRQQSPKSAKEKEKECHTKGQDKQEECEKLESQGCVFNKDGKD
GEKCTLKKEVETELEKATTSEEGKDGKATNSTGSNSFVINKAPLLLAFLLF

Domain combination: A2

GeneDB Blast results N-terminal domains

>but4
MPKATYALVVQTLLLVSKVHQVQPTA
GDAIKKKYWTALCDIAVDADKIAAKALHNLQQPATPGAESLRNLLRALVYNLA
NNTEPTSPGERMIWSHFAEEASAAIDFYKGDKPAKLITAVRDAARANGAILDWINTQHKV
STSSHGCLSGGDSGEAPAAGSAGMATIDAKCVPNWTAVTAEAAATTAVGLQGLQGTLGGQ
KGEGELANGGHGCNSNSANSAVKLLNGGTGTSVAGQSPTLVAGIFKLTGTSMAMDDLSNV
ATKQTTHPYVYHIVQAVKTAGETLATEDVSTIDRAKTSNSFKSVARVHLLGTKATDSRRD
TEIPDKIQAVFGSPDKYTAIYSKNVDEMPLTNELTHDPNLKKLGDITDINDLMRLYFFFL
DRLKSHVKDLTAQLEEAKRQQSPKSAK

>Tb92703
MAKATYALVVQTLFLVSKVNQVQPTA
GDAIKKKY*KALCDIAVYADNIAAKALSSMQEPA
TPCRKALQNLLRALVYNLANSTEQATPGEKMIWSHFAEQTGTTIEFYNGDNPAKLITAVR
DAARAGGAILDRIETQHTISTITHDCLSPNDAGGPPATGNTNLQSMEAKCVSDWTAVKPK
KEATVAVSHVGLQGELSRNNAHSELSNGDHKCNSNSADIAVKLLHGDTGTSLAANNPKLV
AEIFKLTDNGITTEGLANVAAKQQTNPYVYHIVQAVKASEETITLEAVSTLYKAKPSNSP
KSVARVHFLGTIHTDTTRDTEVPDKIQTAFGSPEEYTAIYSRNVDEMPLTKELTHDPNLK
KLSDITDINDLMRLYFFFSDRLKSQVKDLTGQLEEAKRQQAPKSAE

>TbgChr3
MPKATYALVVQTLLLVSQVQQVQPTA
GNAIKKKY*KALCDIAVDADKIAAKALHNLQQPA
TPGAASLRNLLRALVYNLANNTEPTSPGERMIWSHFAEEASAAIDFYKGDKPAKLITAVR
NAARTNGAILDWINTQHKVSTSSHGCLGADNTVTNLVDKDTAMASAAPNCVASWAEVTAN
NAGAATVSAQGFTGVLGTAVPHGTLANTDKKCNSNSGHTAIKLLNDATGTNVGPAEPKLV
AGIFKLTDNGIATEGLRNVDENKKTTHPYVYHIVQAVKTSEETITLEDVSTLDKAKTSNS
FKGVARVHLLGTKATDGSRDTEVPDKIQTAFGSPEEYTAIYSRNVDEMPLTKELTHDPNL
RKLGEITDINDLMRLYFFFSDRLKSQVKDMTGQLEEAKRQQAPKSAK

NCBI blast 2 sequence scores for N-terminal domains excluding N-terminal signal sequences

Tororo v Tbg: 74%
Tororo v Tbb 927: 67%
Tbg v Tbb927: 68%

CLUSTAL W (1.83) multiple sequence alignment

```
but4            MPKATYALVVQTLLLVSKVHQVQPTAGDAIKKKYWTALCDIAVDADKIAAKALHNLQQPA 60
TbgDal          MPKATYALVVQTLLLVSQVQQVQPTAGNAIKKKY-KALCDIAVDADKIAAKALHNLQQPA 59
Tbb927          MAKATYALVVQTLFLVSKVNQVQPTAGDAIKKKY-KALCDIAVYADNIAAKALSSMQEPA 59
                *.***********:***:*:*******:****** .******* **:****** .:*:**

but4            TPGAESLRNLLRALVYNLANNTEPTSPGERMIWSHFAEEASAAIDFYKGDKPAKLITAVR 120
TbgDal          TPGAASLRNLLRALVYNLANNTEPTSPGERMIWSHFAEEASAAIDFYKGDKPAKLITAVR 119
Tbb927          TPCRKALQNLLRALVYNLANSTEQATPGEKMIWSHFAEQTGTTIEFYNGDNPAKLITAVR 119
                **   :*:************.** ::***:********::.::*:**:**:*********

but4            DAARANGAILDWINTQHKVSTSSHGCLSGGDSGEAPAAGSAGMATIDAKCVPNWTAVTAE 180
TbgDal          NAARTNGAILDWINTQHKVSTSSHGCLGADNTVTNLVDKDTAMASAAPNCVASWAEVTAN 179
Tbb927          DAARAGGAILDRIETQHTISTITHDCLSPNDAGGPPATGNTNLQSMEAKCVSDWTAVKPK 179
                :***:.***** *:***.:** :*.**. .::    .  .: : :  .:**..*: *..:

but4            AAATTAVGLQGLQGTLGGQKGEGELANGGHGCNSNSANSAVKLLNGGTGTSVAGQSPTLV 240
TbgDal          NAGAATVSAQGFTGVLGTAVPHGTLANTDKKCNSNSGHTAIKLLNDATGTNVGPAEPKLV 239
Tbb927          KEATVAVSHVGLQGELSRNNAHSELSNGDHKCNSNSADIAVKLLHGDTGTSLAANNPKLV 239
                  .:.:*.  *: * *.    .. *:* .: *****.. *:***:. ***.:.  .*.**

but4            AGIFKLTGTSMAMDDLSNVAT-KQTTHPYVYHIVQAVKTAGETLATEDVSTIDRAKTSNS 299
TbgDal          AGIFKLTDNGIATEGLRNVDENKKTTHPYVYHIVQAVKTSEETITLEDVSTLDKAKTSNS 299
Tbb927          AEIFKLTDNGITTEGLANVAA-KQQTNPYVYHIVQAVKASEETITLEAVSTLYKAKPSNS 298
                * *****...:: :.* **   *: *:***********:: **:: * ***: :**.***

but4            FKSVARVHLLGTKATDSRRDTEIPDKIQAVFGSPDKYTAIYSKNVDEMPLTNELTHDPNL 359
TbgDal          FKGVARVHLLGTKATDGSRDTEVPDKIQTAFGSPEEYTAIYSRNVDEMPLTKELTHDPNL 359
Tbb927          PKSVARVHFLGTIHTDTTRDTEVPDKIQTAFGSPEEYTAIYSRNVDEMPLTKELTHDPNL 358
                 *.*****:***  **  ****:*****:.****::******:********:********

but4            KKLGDITDINDLMRLYFFFLDRLKSHVKDLTAQLEEAKRQQSPKSAK 406
TbgDal          RKLGEITDINDLMRLYFFFSDRLKSQVKDMTGQLEEAKRQQAPKSAK 406
Tbb927          KKLSDITDINDLMRLYFFFSDRLKSQVKDLTGQLEEAKRQQAPKSAE 405
                :**.:************** *****:***:*.*********:****:
```

Buteba 6

MYALMLQLTAVLWLVTQTQAANIVGGANRPDHAALCKFITMAAREVEVPEIPALPTDEY
NYIHMVNFSTAPEDWQNMFFEDKASKKAHADPKAAGATGRGFEANWDQWQKTALSHLDA
TTADAKKQAGVVQLSDTEAALVKPQLAHLAALADELITELSGLQPGPGSTTASSAKQLL
TTAAYGEETVPKPNPNPEKVFGAALNGNRDAICEATSTGPQVATTLATLACVCHKGNAN
TVDPPVCTDQAKGSENWQDADPAATALTGPNLAAIAKSCAKESKEKVTAAELATAITDL
DKLIHKGATDAYLGSFVSTKCNGSSGNGVCVKFASYNTKPSSLVNKLTWIPQLNDLVTK
LMDLERKKIRAEQLVERIKSLKKETTVVIEVAKATASSMEKFETKTNHQKQTSEATSRC
STHNTNTTCTQNKCKWDGTTEDKGKCVADESKGATQTNTAGTGAAGTTTEKCKDKKKDD
CKDGCKWEGETCKDSSILATKKFALSVVSAAFAALLF

Domain combination: B1

GeneDB Blast results N-terminal domains

Tbg closest match >gamb1360f04.p1k_2

>But6
MYALMLQLTAVLWLVTQTQA
ANIVGGANRPDHAALCKFITMAAREVEVPEIPALPTDEYNY
IHMVNFSTAPEDWQNMFFEDKASKKAHADPKAAGATGRGFEANWDQWQKTALSHLDATTAD
AKKQAGVVQLSDTEAALVKPQLAHLAALADELITELSGLQPGPGSTTASSAKQLLTTAAYG
EETVPKPNPNPEKVFGAALNGNRDAICEATSTGPQVATTLATLACVCHKGNANTVDPPVCT
DQAKGSENWQDADPAATALTGPNLAAIAKSCAKESKEKVTAAELATAITDLDKLIHKGATD
AYLGSFVSTKCNGSSGNGVCVKFASYNTKPSSLVNKLTWIPQLNDLVTKLMDLERKKIRAE
QLVERIKSLKKETTVVIEVAKATASSMEKFETKTNHQKQTS

>Tb11.v4.0034
MYAIMLPMTIVLWLVTKTEG
TDIVGGANRQEHAALCKFVAMAPREVEIPTIPAMPEDDLD
YIHMVNFSAATGSWQEMFYSDKAAKKTHDNPKSAGQDGRGFEENWPRWTKIAAKKLEATT
GGQKNQAGIMELTEDQETLARDHLKHIRTRAHELAKELQSIQPPSDAPKDNTAKETIATA
VYGENAKPASNPDPSKVFTGTTTGTRDSVCKAAGGNGNPATALEALTCVCFRGTNNVDPA
VCVQAADGGTWEAASTFATGVTAANLGKLAKSCPFDAGQITGTEILAAIANLNDLIHKDT
SNAYLGQFASSKCDGNKAQGVCLEFSGFASKAQPIVEQLTWIPPLKNLALGLQTLESNKQ
RGLQIIEQIKKLKKDAVVIVDSAKATAAALEKIKGGTQKQHTIG

NCBI blast 2 sequence scores for N-terminal domains excluding n-terminal signal sequences

Tororo v Tbg: N/A
Tororo v Tbb 927: 46%
Tbg v Tbb927: N/A

Buw1

MSMKQGGAALVLLIMTIKRVNALVADESEAAHRAMCALTEAASATFTAPSGTEQADELNDK
IQAANMSVADAHWQALFDSEAGSKPYDETTGDNRTIADSLGGQEKWDGWRKAFANIKALNI
GTATKGEYPKITNEVDRKIARQQLRPIAAQAIKLAAAMKPLKAFLSDGKINKINENLRKAL
YGGDGDLQTPTISKSFGDQGDTWNNLCQGSAKRKSIAGDFFCICTGAAAATKQCSAAYSNT
EHSNQPSINTGWTALQKSCGKREQSKATVESIYAAVTQWRVALNQKGSGSENSVWLGKSSG
TGTECAGTDGNTCVDYSGFFKANSGADLATLPWLSALSEAAAKLRQAAEAAAAVKSLQTQM
QALQGATVEIYSAAASGVLAKEMTIAIPTAPPTQHELQPQTQKKQAELAETECNTAGDNKA
ECEKKTGCTYDEAKDKGKRCKLSEEAKKVADGTAKDAKTNTTASNSFVIHKAPLLLAFLLF

Domain combination: B2

GeneDB Blast results N-terminal domains

>Buw1
MSMKQGGAALVLLIMTIKRVNALVAD
ESEAAHRAMCALTEAASATFTAPSGTEQADELND
KIQAANMSVADAHWQALFDSEAGSKPYDETTGDNRTIADSLGGQEKWDGWRKAFANIKAL
NIGTATKGEYPKITNEVDRKIARQQLRPIAAQAIKLAAAMKPLKAFLSDGKINKINENLR
KALYGGDGDLQTPTISKSFGDQGDTWNNLCQGSAKRKSIAGDFFCICTGAAAATKQCSAA
YSNTEHSNQPSINTGWTALQKSCGKREQSKATVESIYAAVTQWRVALNQKGSGSENSVWL
GKSSGTGTECAGTDGNTCVDYSGFFKANSGADLATLPWLSALSEAAAKLRQAAEAAAAVK
SLQTQMQALQGATVEIYSAAASGVLAKEMTIAIPTAPPTQHELQPQTQKKQAE

>Tb927_11
MSIQQGAAALVALIMLATDRVKAVAD
ESEAAHRAMCALTEAASATFTTPAGSPEAEGLS
DKIQAANMSVADARWQAFFDSEAGSKPYDETTGDNRTLADKLGGKEKWDGWRKAFANIKV
LNIGTKPQGEYPKINSEVDRKLARQQLRAVAAQATQLERTMKPLKAFLSDDKSNKINENL
RKALYGGDGELSTPTLGKSFGANGESWAGLCATKANRKSIAGDFFCICTGADATTKQCSG
AYSNTAHDSQPSINTGWTALQQSCGKREQSKATAGTIYAAVAQW*AALKQKASGSDNSVW
LGKSSSTGTACAGTDTNTCIDYSDFFKKSAGADLATLPWLSALSDAAA
QLKQAEEAAAAVKSLQAQIQALQGASVEIYSAAASGVLAKEMPIAIPTAPPTQQ
RLQPQTQKKQAE

>gamb217f04
RQASTALRTLIMLTTDRVKADAD
DTEAVHRAMCALTEAASVTFTPPAAVAEPAGLNDKI
QAANMSVADARWQALFDSEVGSKPYDETTGDNRTLADKLCGKEKWDGWLQAVANIKALNI
GTKPEGEYPKINSEVDRKISRQQLRPIAAQATKLAIAMK*LKAFLSDDKINKINENLRKA
LYGGDGELSTPTLGKSFGANGES*AGLCATKANRKSIAGDFFCICTWQNAVTK*CSGAYS
HTAHNDQNNINTGWTALQKSCGKREQSKASVASIYAAIAQWRAALKQKNKGGSDNSVWLG
ASDNTGATCAGTDTNTCVDYSDFFK
KTGGIDLAT*HRLKALTAVEQ*LKQAEETAAGVMSLQVQMQAVQGASVEINSAAASG
VLSKEMPITVPTARSQSTEINYKHSKNK
Reading frame adjusted after DFFK

NCBI blast 2 sequence scores for N-terminal domains excluding n-terminal signal sequences

Tororo v Tbg: 69%
Tororo v Tbb 927: 80%
Tbg v Tbb927: 78%

```
CLUSTAL W (1.83) multiple sequence alignment


Buw1            MSMKQGGAALVLLIMTIKRVNALVADESEAAHRAMCALTEAASATFTAPSGTEQADELND 60
Tbb927          MSIQQGAAALVALIMLATDRVKAVADESEAAHRAMCALTEAASATFTTPAGSPEAEGLSD 60
TbgDal          ---RQASTALRTLIMLTTDRVKADADDTEAVHRAMCALTEAASVTFTPPAAVAEPAGLND 57
                   :*..:**  ***  .      **::**.************.***.*:.  :.  *.*

Buw1            KIQAANMSVADAHWQALFDSEAGSKPYDETTGDNRTIADSLGGQEKWDGWRKAFANIKAL 120
Tbb927          KIQAANMSVADARWQAFFDSEAGSKPYDETTGDNRTLADKLGGKEKWDGWRKAFANIKVL 120
TbgDal          KIQAANMSVADARWQALFDSEVGSKPYDETTGDNRTLADKLCGKEKWDGWLQAVANIKAL 117
                ************:***:****.**************:**.* *:****** :*.****.*

Buw1            NIGTATKGEYPKITNEVDRKIARQQLRPIAAQAIKLAAAMKPLKAFLSDGKINKINENLR 180
Tbb927          NIGTKPQGEYPKINSEVDRKLARQQLRAVAAQATQLERTMKPLKAFLSDDKSNKINENLR 180
TbgDal          NIGTKPEGEYPKINSEVDRKISRQQLRPIAAQATKLAIAMK-LKAFLSDDKINKINENLR 176
                **** .:******..*****::*****.:**** :*  :** *******.* ********

Buw1            KALYGGDGDLQTPTISKSFGDQGDTWNNLCQGSAKRKSIAGDFFCICTGAAAATKQCSAA 240
Tbb927          KALYGGDGELSTPTLGKSFGANGESWAGLCATKANRKSIAGDFFCICTGADATTKQCSGA 240
TbgDal          KALYGGDGELSTPTLGKSFGANGES-AGLCATKANRKSIAGDFFCICTWQNAVTK-CSGA 234
                ********:*.***:.**** :*::  .**  .*:*************   *.** **.*

Buw1            YSNTEHSNQPSINTGWTALQKSCGKREQSKATVESIYAAVTQWRVALNQKG-SGSENSVW 299
Tbb927          YSNTAHDSQPSINTGWTALQQSCGKREQSKATAGTIYAAVAQW-AALKQKA-SGSDNSVW 298
TbgDal          YSHTAHNDQNNINTGWTALQKSCGKREQSKASVASIYAAIAQWRAALKQKNKGGSDNSVW 294
                **:* *..* .*********:**********:. :****::** .**:**  .**:****

Buw1            LGKSSGTGTECAGTDGNTCVDYSGFFKANSGADLATLPWLSALSEAAAKLRQAAEAAAAV 359
Tbb927          LGKSSSTGTACAGTDTNTCIDYSDFFKKSAGADLATLPWLSALSDAAAQLKQAEEAAAAV 358
TbgDal          LGASDNTGATCAGTDTNTCVDYSDFFKKTGGIDLATH-RLKALT-AVEQLKQAEETAAGV 352
                ** *..**: ***** ***:***.*** ..* ****   *.**: *. :*:** *:**.*

Buw1            KSLQTQMQALQGATVEIYSAAASGVLAKEMTIAIPTAPPTQHELQPQTQKKQAE 413
Tbb927          KSLQAQIQALQGASVEIYSAAASGVLAKEMPIAIPTAPPTQQRLQPQTQKKQAE 412
TbgDal          MSLQVQMQAVQGASVEINSAAASGVLSKEMPITVPTARSQSTEINYKHSKNK-- 404
                 ***.*:**:***:*** ********:***.*::*** . . .:: : .*::
```

Buw2

MTTPGKIVIAYVITTAITTYVSSANAALKDTTWKSLCGLEEELRKLPSVAATTLERQETSVN
AYKITGLKTLLYGHTLGDSKDAAAIASLGLTLLKKADEEQSSKHTGTTTAIKALTSSQELVG
RIHEGIEILAIATHDSVWCLGNTAGTADAAGERTGAGCKGKIHDLTTTAANLGDDVIDSDGY
KTIQDITDGTGVADSSKCPFTAVAANHQTWGAGKANAKMIDGMLTFTTAEQVTRSGFTKKRA
AKDRLTDKLSVTVHADVMALKEAYRNTEIRAGSSALKAAAEDNQLTPTLTRLLQRAPYNMPA
ADADAEAKNLVKTKFSTTGSTLEDLWKQIKDASVVDIMKETEEATTIEKIQQLETLRATIEL
YAASDKGKLAKLKKELEQAQADKNGAKVSKTTEECNDHKELGPCQKAGCKFDNSKNDGEKCF
PDPEAKTDKKGREDGKTTTTSTTGSNSVLINKAPILLAFLLF

Domain combination: A2

GeneDB Blast results N-terminal domains

Tbg closest is >gamb249a02.p1k at 31%

N-terminal domains

>Buw2
MTTPGKIVIAYVITTAITTYVSS
ANAALKDTTWKSLCGLEEELRKLPSVAATTLERQETS
VNAYKITGLKTLLYGHTLGDSKDAAAIASLGLTLLKKADEEQSSKHTGTTTAIKALTSSQ
ELVGRIHEGIEILAIATHDSVWCLGNTAGTADAAGERTGAGCKGKIHDLTTTAANLGDDV
IDSDGYKTIQDITDGTGVADSSKCPFTAVAANHQTWGAGKANAKMIDGMLTFTTAEQVTR
SGFTKKRAAKDRLTDKLSVTVHADVMALKEAYRNTEIRAGSSALKAAAEDNQLTPTLTRL
LQRAPYNMPAADADAEAKNLVKTKFSTTGSTLEDLWKQIKDASVVDIMKETEEATTIEKI
QQLETLRATIELYAASDKGKLAKLKKELEQAQADKNGAKVS

>Tb927chr5
MTTTGKIVIAYVIATAVTTYVAG
TLAALKKNVKKPLCGLEEELRMLPSVAATALKRQEAS
SNAYKITGFKTLLYGHTLDDSKDTAAITALGQTLLKKADDEQNAKHATTATAIKALTFSQ
ELVGRIHEVIEILATATHDSVYFLGNSGATSNAAAERTTAGCKGKIHDLTTTADNIDDDI
LDENGYKTITDVENTNGVGDSNKCPFTAQAASHQTWGAGTADAEMIDGMLTCTTAEQVAR
SGFTKVGTANNRLTDALSITVHADTARPKSAYRETKIKGKAAIEQAAEDSQLTPTLKRLL
QRAPYNMPAADANSEADNLVKTKFSTGTPLEKL*KEIKDASVVDIMKETEEPTTIEKIQ
QTETLRVTIEFYAASDKEVLAKLKKELDQAQAAKNGGKVS
(Reading frames adjusted)

NCBI blast 2 sequence scores for N-terminal domains excluding n-terminal signal sequences

Tororo v Tbg: N/A
Tororo v Tbb 927: 74%
Tbg v Tbb927: N/A

Do1

MIPATGSPAALAVPQQKQASRYLIVVVAAAMTTKVATAALEEGANAPAF
HNLCTILAAADTDLAIGQETTTDKDKLLDTIHQLNHTFSDQSWQNEFKTAKGPGNWRDTL
PEKYKGDAKWQEMYPTWLKAAKEAETDEENTAVKKIGAEGLSEAQKLLLRPTVQQLAARA
HDIKNQLQSLAKLEQSDDAKGASKLLKEAAYGSATKTRENTAHTDIFASTCTSTATNCCK
DDSSQKPPPTAAGVAMCLCAADGTKSKMCVNPQTALSNWNDPQSNHVAEWAKLRVYCSEK
LGGDRPEEAINNALESLMANLHFNGNKLYLGAWQTGACRSGSGNAFCVSYTSDASKDASA
VNKQPWVEKTRRAVAALRNSRQATAEAKQLKRQLTTIVAELAAAAQAAKNMNTKTINTEK
TAPTSTKDLNANCSKHKDSKTTCENTGKCEWKGKTETDGTCEVDESKVTTQTNVAKAGTD
SKTNTTGSNSFVIHTAPLLLAVLLF

Domain combination: B2

GeneDB Blast results N-terminal domains

>Do1
MIPATGSPAALAVPQQKQASRYLIVVVAAAMTTKVATA
ALEEGANAPAF
HNLCTILAAADTDLAIGQETTTDKDKLLDTIHQLNHTFSDQSWQNEFKTAKGPGNWRDTL
PEKYKGDAKWQEMYPTWLKAAKEAETDEENTAVKKIGAEGLSEAQKLLLRPTVQQLAARA
HDIKNQLQSLAKLEQSDDAKGASKLLKEAAYGSATKTRENTAHTDIFASTCTSTATNCCK
DDSSQKPPPTAAGVAMCLCAADGTKSKMCVNPQTALSNWNDPQSNHVAEWAKLRVYCSEK
LGGDRPEEAINNALESLMANLHFNGNKLYLGAWQTGACRSGSGNAFCVSYTSDASKDASA
VNKQPWVEKTRRAVAALRNSRQATAEAKQLKRQLTTIVAELAAAAQAAKNMNTKTINTEK
TAPTSTK

>Tb09.244.1110
MIALAFASVPKLRANAAVAAGEN
AALFRGLRTILSAAETKITLGSETTAEDPNIGAIQQL
NLTFSKPTWKAVFKTKPGEGNWRDEIPDTHKENKQWKALYPAWLKAAQAEEEDTEPNVLK
EVGAAKLSKHQKIYFGSEVAKVAAQAAKIAENLQELKTHERSRDAESAHKLLKTAAFGGA
DKARTRLKHTDMFTSGCAADPQVCCKDDTNTGKPPITVAGVIMCVCAADANEPKACINPQ
TALTTWANPSTATATEWPKISAYCTANSGQEINVDSIDAALDRVLATGRFDTKKLYIGAA
NSGACRSGSANGFCTTYTAASATDTSKLNSAPWLANVRKATRALRLSKEATEEAKRLKTQ
IATLYAGLSGTAAAATTQIPEPPALDKGTTVARV

>gamb261a02.p1k
MIALAFASVPKLRANAAVTAGEN
AALFRDLCTIVSGAKS
TATIGSETTTLDPTVGSIHQLNLTFSDPAWKAVFKTKPGKENWQEEIPDAHKENAQWKAL
YPTWLAAAKADEQDTELKVLKAVGADKLKPHQKAYFGSEVAKVAAQVSKIAETLQELKAE
ERSTDAESAQKLLRTAAFGGPDKTRSGLTHIDMFTSGCAANQAVCCKDDDGTGKPPITVA
GVIMCVCAHDGNLGTACINPATALATWADPSAATQAEWTKISAYCTANSGQEINVDSIDD
ALGRVLATARFDSSKLYIGAANGGACRSGSANGFCTTYTAASATDTSKLTAAPWVANVRK
ATRALRLSKKATEDAKRLKAQLATLYAGLSGAAAAAARIPEPPALDKGTTVARLT

NCBI blast 2 sequence scores for N-terminal domains excluding n-terminal signal sequences

Tororo v Tbg: 44%
Tororo v Tbb 927: 45%
Tbg v Tbb927: 77%

Do2

MSHARYVFLATFITAQMASFSAG
QAISTLANKHEYRELCKLLALARTGITLQDLSSPPTEDINLVMDINMSLSEVSWQNMFVKDKSKGEWHANAAE
AKQEGKGYEQSWDKWLNSRQRLKEDAQTTEFKQLKLEDLKPHQKRSLRRHVQAVAEEVAN
EASGFVGGESTEGQLNAAEATKTLREAAYGAGDTTEAKVTAQQAFGAAMTDQARTATCTT
NAQGRAGKSVLATAACLCLKPQEGTQVDGACGMTLDGSGTWRDSAAAPTSADIQNLAKYC
AGDGSTNDPGARIKAALTALATSVVRGTTDAHLGAFKTGNCDGKNTGGICVELKNGARAE
DGGVTQLGWYEKLNTLADKLIRRQQAQTRNKHAKEKIQKLVTTLKAFIKVTKGEAAPEIA
ADAPNAKKGAPGPATSCSSYNTNATCHQNNCKWEENASDKSKGTCKPKTETETPPAGAGT
GAAGASAEDKKCSNKKKQEDCKDGCKWEGTECKD
SSILATKKFALSVVSAAFVALLF

Domain combination: B1

GeneDB Blast results N-terminal domains

Tbb 927 present in reads
>tryp_XI-346e09.p1k rev comp
reading frame adjusted by inclusion of n at 316
Cannot extend at 3′ end

Tbg:
>gamb1337e11.p1k_5 33% identity

>Do2
MSHARYVFLATFITAQMASFSAG
QAISTLANKHEYR
ELCKLLALARTGITLQDLSSPPTEDINLVMDINMSLSEVSWQNMFVKDKSKGEWHANAAE
AKQEGKGYEQSWDKWLNSRQRLKEDAQTTEFKQLKLEDLKPHQKRSLRRHVQAVAEEVAN
EASGFVGGESTEGQLNAAEATKTLREAAYGAGDTTEAKVTAQQAFGAAMTDQARTATCTT
NAQGRAGKSVLATAACLCLKPQEGTQVDGACGMTLDGSGTWRDSAAAPTSADIQNLAKYC
AGDGSTNDPGARIKAALTALATSVVRGTTDAHLGAFKTGNCDGKNTGGICVELKNGARAE
DGGVTQLGWYEKLNTLADKLIRRQQAQTRNKHAKEKIQKLVTTLKAFIKVTKGEAAPEIA
ADAPNAKKGA

>Tbb927
MPHVHYVFSATFISGEMVWHSTY
QTIRTLANKHKYRELCKILALARTDITLQDLSSPPHE
DINLVMDITMSLSDPSWQDMFVKDKSKGEWHANAADANQKGKGYEDQWADWLAARKRIKK
LSKXTAFKGLNLAELKDYQKTNVRQHVQAVAEQVATAASNFVPATSEDAKLTSDDVTPTL
RQAAFGDKAATEGGVTIQQAF

NCBI blast 2 sequence scores for N-terminal domains excluding N-terminal signal sequences

Tororo v Tbg: N/A
Tororo v Tbb 927: 64% over available sequence
Tbg v Tbb927: N/A

Kinu1

MDYKDCYGYLAALTFVVSLCSG
APLTDNDPVAEQISDLCK
EKFYVETLIQNLKQRVQSTADSIKDMEKLKATWEKAAAATSDAAKKCLFSALEHKADAEL
LRELPKIEEATEAVTTAQIALSEHVGMLGATITLAKTKLDSGSSNHGNADTGSIRIALSR
TTATTDLCNEPATISDIKPGVSEIQPGKLFKLKLTKPTDLHKHMFVDWLTIGGLKSCTAH
TSYDQNFDGALSGCQYSASGTAVATQASTKPPYATAAVTLFKNNDPEQQCEVTDLPAGGA
ADKHKKLQHSLCRALQLGNVKGHSLRQLDGAALQSDHVVANTIRNCDPVFQKLTTATDGE
GTTELKKYIKEAYGSSASEFAEKFITNAEKLQMTLRLNDKIETKDLSAVSPGEQTAAALS
HIQGLHNKRELEAGKKSTSAAAVDPQKSEDCKGEKDETKCNKKDGCEFKDGECLAKVTTA
AGTDGKTNTTGS
NSFVIHKTPLLLAVLLLA

Domain combination: A2

GeneDB Blast results N-terminal domains

Tbg – contig cannot be assembled further in 3′ direction
>gamb606g06.q1k
>gamb1287f01.q1k

>kinu1
MDYKDCYGYLAALTFVVSLCSG
APLTDNDPVAEQISDLCK
EKFYVETLIQNLKQRVQSTADSIKDMEKLKATWEKAAAATSDAAKKCLFSALEHKADAEL
LRELPKIEEATEAVTTAQIALSEHVGMLGATITLAKTKLDSGSSNHGNADTGSIRIALSR
TTATTDLCNEPATISDIKPGVSEIQPGKLFKLKLTKPTDLHKHMFVDWLTIGGLKSCTAH
TSYDQNFDGALSGCQYSASGTAVATQASTKPPYATAAVTLFKNNDPEQQCEVTDLPAGGA
ADKHKKLQHSLCRALQLGNVKGHSLRQLDGAALQSDHVVANTIRNCDPVFQKLTTATDGE
GTTELKKYIKEAYGSSASEFAEKFITNAEKLQMTLRLNDKIETKDLSAVSPGEQTAAALS
HIQGLHNKRELEAGKKSTSAAAVDP

>Tb09.244.0250
MDYKDCYGYLAALTFVVSLCSG
APLTDNDPVAEQISDLCKEKFYVETLIQNLKQRVQSTA
ASIKDMEKLKATWEKAAAATSDAAKKCLFSALEHKADAELLRELPKIEEATEAVTTAQIA
LSEHVGMLGATITLAKTKLDSGSSNHGNAGTGSIRIALSRTTATTDLCNEPATISDIKPG
VSEIQPDKLFKLKLTKPTDLHKHMFVDWLTIGGLTSCTAHTSYDQNFDAALSGCNYKTAA
TAEAKQATTKPTYGTTEVTLFKNNDPEQQCEVTDLPAGGAADKHKKLQHSLCRALQLGNV
NGRSLRQLNGAALQSDHVVANTIRNCDPVFQKLTTATDGEGTTELKKYIKEAYGSSASEF
AEKFITNAEKLQMTLRLNDKIETKDLSAVSPGEQTAAALSHIQGLHNKRELEAGKKSTSA
AAVDP

>gamb1287f01
MDYKDCYGYLSALTFVASLCSG
APLTDNDPVAEQISDLCKEKFYVETLIQKLKQRVQSTA
ASIKDMEKLKATWEKAAAATSDAAKKCLFSALEHKADAELLRELPKIEEATEAVTTAQIA
LSEHVGMLGATITLAKTKLDSGSSNHGNANTGSIRIALSRTTATTDLCNEPATISDIKHG
VSEIKADKLFKLKLTKPTDLHKHMFVDWLTIGGLTSCTAHRSYDQNFDAALSGCQYSASG
KAVATQASTKPTYATAAVTLFKNNDPEQQCEVTDLAPGDSEDK

NCBI blast 2 sequence scores for N-terminal domains excluding N-terminal signal sequences

Tororo v Tbg: over 93% over available sequence
Tororo v Tbb 927: 95%
Tbg v Tbb927: 91% over available sequence

Maw-ero 1

MHQVLLAAIVLLSPMTKHASG
AIGAGANVAVFEALCGLISLSK
STITPPPQVKTHLTELATLKKLNMSVSESSWRNLFHKNGAKDQYNDQVPQGVAKGDDWDA
KWSGWKEAEKALEKADTDPDLKAAGLTKPDTKLASAARHAIAALAESAETTAAGALEPPD
VEEEIGGSKLQTALRTAAFGADVASEQQATIQAAFGGDATQTRSTACEASTAENKAQTVL
AAIACVCMDINSNAVNGACHHKHVAAADWSGTSISLPNLQALAAYCPPRPTKPITAAELR
AAIAAVENKITVFGTDGYLGAAVTSCDGQTANGVCVKFTGYKTNPQTSTANLPWIGKLKT
LAAKLEQREKANQKAQEITSIFKVDIKKVKFIVLEAKQKVEGQTTNTLSAAHAGGQSNVA
GKPCEEQTSNTTCRADNNCKWDSKEESEGNFCKPKDGEEQTKTAGAGGDEAAAAKEEGKK
CSDKKKEEDCKSPDCKWEGTECKD
SSIIVTNQFALSMVSAAFVALLF

Domain combination: B1

GeneDB Blast results N-terminal domains

Tbb 927 best hit >Tb10.v4.0061 33% identity

>Mawero1
MHQVLLAAIVLLSPMTKHASGAIG
AGANVAVFEALCGLISLSK
STITPPPQVKTHLTELATLKKLNMSVSESSWRNLFHKNGAKDQYNDQVPQGVAKGDDWDA
KWSGWKEAEKALEKADTDPDLKAAGLTKPDTKLASAARHAIAALAESAETTAAGALEPPD
VEEEIGGSKLQTALRTAAFGADVASEQQATIQAAFGGDATQTRSTACEASTAENKAQTVL
AAIACVCMDINSNAVNGACHHKHVAAADWSGTSISLPNLQALAAYCPPRPTKPITAAELR
AAIAAVENKITVFGTDGYLGAAVTSCDGQTANGVCVKFTGYKTNPQTSTANLPWIGKLKT
LAAKLEQREKANQKAQEITSIFKVDIKKVKFIVLEAKQKVEGQTTNTLSAAHAGGQSN

>gamb1327e07
MISVIRALAVILSPIALVGTVDGTKS
TGANVAPFEALCGIIALAKSAVKVPAVTNSHIEQ
MNKVRKLNMSVTDPTWRIIFHKNGKPNEYNDNVPRGIVSGPDWAEQWKQLKEAEQKLENE
AEGTDFKNSHLNTATPDVKRIIRPRIAAIAARAAIKVKSIKEDPQTTEALTDSKVQTLLR
KAVFGDDLADEQGATHQSLFGGTSLSNRGTVCEASTQANKAQTAGAVIARLFLDSNSDIV
ADIFHHAATGGAHWNAGNPDLGQIQAHIARCRPAVSELLTYDKLIGLITAITTRISLWSG
DGYLGAVTSACSGTTATGSCVKFSGYKANPDQATSALQCLGHLRRLAEQLKDREDMNKKT
EEATRILK

NCBI blast 2 sequence scores for N-terminal domains excluding n-terminal signal sequences

Tororo v Tbg: 42% over available sequence
Tororo v Tbb 927: N/A
Tbg v Tbb927: N/A

Mul 1

MKRKQLVLLTLVAAVAPASG
ALDDNAAEFRALCDVYNLYQQKAKMTEDSNSDSMQPTLERLIKANLSTATETYFNNKDGAFTKEGKTDEQSIQKWREEA
NKMVSEEDTKTKAKKSTRMPPSEARSAANKRIMELHRDSENVLEEYKAAVAIAKAARQEAAAYLTYAIFGDNNTDLTED
KIPSTTKQMCGNAAGGHADVGQGIAHALTCLCSVTTGANNECAKGLTTTGNQGSQTATQTAENWKTIAEACSKRKSSMT
PTPELLEAITVGVEAHLGRQPNGAADGDNAYVLGKLGASSVCDMGTNKACINYKTQMTTGTKDVAWQRNLRKAAMELRK
AADLDRQAKLHKIHLQHNDEAAATILLTAELTSKAASTSTLSPSPRSNQQSAPGEESKCKPQNNSAEECPSEHCNYDTK
TKECKPKKTGSETPAAAGTGDASSGVDCSKHQTQQACEAENKDVKQGQKAVCGWIDYVEGTGKLPKPECRS
SSFLLNEKFALSVVSAAFAALLF

Domain combination: B2

GeneDB Blast results N-terminal domains

Tbb 927 closest hit >Tb11.57.0037 36 % identity

Tbg closest hit >gamb761b01.q1k_0 30 % identity

N-terminal domains

>Mul1
MKRKQLVLLTLVAAVAPASGALDDNAAEFRALCDVYNLYQQKAKMTEDSNSDSMQPTL
ERLIKANLSTATETYFNNKDGAFTKEGKTDEQSIQKWREEANKMVSEEDTKTKAKKST
RMPPSEARSAANKRIMELHRDSENVLEEYKAAVAIAKAARQEAAAYLTYAIFGDNNTD
LTEDKIPSTTKQMCGNAAGGHADVGQGIAHALTCLCSVTTGANNECAKGLTTTGNQGS
QTATQTAENWKTIAEACSKRKSSMTPTPELLEAITVGVEAHLGRQPNGAADGDNAYVL
GKLGASSVCDMGTNKACINYKTQMTTGTKDVAWQRNLRKAAMELRKAADLDRQAKLHK
IHLQHNDEAAATILLTAELTSKAASTSTLSPSPRSNQQSAP

NCBI blast 2 sequence scores for N-terminal domains excluding n-terminal signal sequences

Tororo v Tbg: N/A
Tororo v Tbb 927: N/A
Tbg v Tbb927: N/A

Mul3

MNKKILILTVFALAAGKNKIQA
TDELPNAPDFAVLCRIVQHAK
AGFKQASEDEKSVASQLAATTAKLAIINDDDETRYLDKNNRTIYRITGDEPKIPKGAEKK
PLKAKLLQLERRAAELKNDYDNTFAEATRQIEQANDELAEAVYGAGAKFEDQGDSSKLIT
NARASSLFGATGTYNKNCGGTNGGGAASTSNVGITLVSDIYCLCIAGTASAKTCDQTTTA
LSHGTLFASTAGTGKEAFDALMAKCGSQPQQTSPSELHALLIAWQSKLGSHFESTNTRDA
ARFIIGRADNPATGCTGAAKQHCVDYKSFLGATPSQDPRWVSKIKSAITKVQTAAEKSLR
LRHSLSLLEVVSDQANLTYTEGLNSQRTQRHNAENNAPTKQTETDETCEKKGTGDGCKDG
CKWDGEGDNKKCVKDLDYKPKQAEGGEKESKTGTTNTTGS
NSFIINKGPLLLTFLLF

Domain combination: B2

GeneDB Blast results N-terminal domains

Tbg contig assembled by hand

>gamb86e09.p1k
>gamb1296h01.q1k

Sequence adjusted to maintain frame by inclusion of n at 170
Cannot be extended

>Mul3
MNKKILILTVFALAAGKNKIQA
TDELPNAPDFAVLCRIVQHAK
AGFKQASEDEKSVASQLAATTAKLAIINDDDETRYLDKNNRTIYRITGDEPKIPKGAEKK
PLKAKLLQLERRAAELKNDYDNTFAEATRQIEQANDELAEAVYGAGAKFEDQGDSSKLIT
NARASSLFGATGTYNKNCGGTNGGGAASTSNVGITLVSDIYCLCIAGTASAKTCDQTTTA
LSHGTLFASTAGTGKEAFDALMAKCGSQPQQTSPSELHALLIAWQSKLGSHFESTNTRDA
ARFIIGRADNPATGCTGAAKQHCVDYKSFLGATPSQDPRWVSKIKSAITKVQTAAEKSLR
LRHSLSLLEVVSDQANLTYTEGLNSQRTQRHNAENNAPTKQT

>tbb927
MNKKILILTVFALAAGKNKIQA
TDELPNAPDFAVLCRIVQHAKAGFKQTSEDEKSAASQL
AAKTAKLAIINDDDETR*LDKNNRTIYRITGKEPKIPTRAETKPLKAKLLEIERRAIELK
TDYDTKSSDSSRQIQQANDDLAEAVYGAGAKFDDQGDTSKLITNGRASNLFGKAGTYAKN
CGGTNGGGTATTSNVGITLVSDIYCLCIAGTATTKTCDQTTTASSHATLFASTAGSGKGS
SDALTAKCGSQPQQTSPSELHALLIAWQSKRGAHFESTNTKDEARFIIG*ATNQATGCTG
ANKQHCVDYKYFLGGTPSQDALWVSKIKSAIGKVQTADEKSLRLRHNLSLLEAISDQANL
TYTESLNSQRTQRDNAENNVPTKQT

>tbg
KFDDQGDTSKLITNGRAASLFGPAGTYAKNCGGTNGGGTATTSNVGITLVSDIYCLCIAG
TATTKTCDQTTTASSHETLFASTAGGGKGASDALTAKCGSQPQQTSPSELHALLIAWQSK
RGAHFESTNTKDEARFIIG-ATNQATGCTGANKQHCVDYKSFLGGTPSQDALWVSKIKSA
IGKVQTADEKSLRLRHNLSLLEAISDQANLTYTEGLNSQRTQRDNAENNVPTKQT

NCBI blast 2 sequence scores for N-terminal domains excluding n-terminal signal sequences

Tororo v Tbg: 85% over available sequence
Tororo v Tbb 927: 83%
Tbg v Tbb927: 96% over available sequence

Mul 4

MTNAKRAFISFKTLFLITTTNLFTAEA
NTAPGDNTAQYKAICMLVNLANKCTKGGQATTTKTDTALVVGAINISLASNDFQQAIDTE
KDWATLPQDKKNKLGGSEEDWQFWKESKRQLKKHQAWIKTFTGQPKTAAQRLATAEMTLR
ARKLYEQAQSASATPATSTAQLCKDALYGQGKTSSDGLAAGHATRANVCSDEAAGTNNKA
GTALFWDVLCLCGGSSTHANTKQACGKGLEASETRTDWTPNTNDQAAGDPLIQHCATFGG
AGHLNAVTLESAWAALQTQIKTDSNTNAGKPLVLGAVDASGAAFTGCTGNKVTNGGQCVQ
YKAKHFSNGETTIPWLSALRRAAEKAKSDEDNAKKAQQVEKELQLLNDTVIAILVGEKTT
TTTAASAEESASKKQQEEAEANCNKIEKDTDCKAKPKCAWDGTAKDPNKKCTLSEEAKKE
AAKEAAEKQTGTDGKTNTTGS
NSFVIKKAPLLLAVLLF

Domain combination: B2

GeneDB Blast results N-terminal domains

Tbg contig assembled by hand
>gamb248h03.p1k rev comp
>gamb381f10.p1k
>gamb1357f09.p1k rev comp
Reading frame adjusted by insertion of n at 1122

>Mul4
MTNAKRAFISFKTLFLITTTNLFTAEA
NTAPGDNTAQYKAICMLVNLANKCTKGGQATTT
KTDTALVVGAINISLASNDFQQAIDTEKDWATLPQDKKNKLGGSEEDWQFWKESKRQLKK
HQAWIKTFTGQPKTAAQRLATAEMTLRARKLYEQAQSASATPATSTAQLCKDALYGQGKT
SSDGLAAGHATRANVCSDEAAGTNNKAGTALFWDVLCLCGGSSTHANTKQACGKGLEASE
TRTDWTPNTNDQAAGDPLIQHCATFGGAGHLNAVTLESAWAALQTQIKTDSNTNAGKPLV
LGAVDASGAAFTGCTGNKVTNGGQCVQYKAKHFSNGETTIPWLSALRRAAEKAKSDEDNA
KKAQQVEKELQLLNDTVIAILVGEKTTTTTAASAEESASKKQQE

>Tb927
MTNAKRAFIRFKTLFLLTAANLFTAEA
NTAAGDNTAQYKAICMLVNLATKCTKGGQEATT
KTDTALVVGALNISLAGSDFQQAIDTEKEWATLPQANKNKLGGSEEDWQFWKESKKQLKH
HQTWIKTFTAQPNTAAQRLATAEMAKRAKKLYAQAQTAEQTPAPTAAQLCKDSLYGHGAA
NSDGLKAGHSARENVCSEQAGGPNNKAGTALFWDLLCLCAGDGQQETTKKACGKGLEGSE
TRTAWAPDGNDKEAGDPLIEHCAKFGGAGHLTAVTLEAAWAALQTQIKTDSSTNAGKALV
LGAVDSVSGGAFAGCTGNKGTNGGQCVQYKPKHFANGETTIPWLTALRRAAEKVKNDEDN
AKKAQQVEKELQLLNDTVIAILVGEKTTAT-AAPVEESASKKHQE

>Tbg
MTNAQRAFISFKTLFLITTTNLFTAED
NTAPGDNTAQYKAICMLVNLVKKCTKGGQANTT
KTDTALVVGAINISLAGSDFQQAIDTEKELATLPQDNKNKLGGNKADWKFWKESKKQLKN
HQTWIKTFAAQPKTAAKRLATVEMAIRARNLYAEANQASATPAAYAAQLCKEALYGQGQT
SSYGLKAGHATRENVCSEEAAGTSNKAGSALFWDVLCLCGGSSTHANTKQACGKGL-ASE
TSTDWTPNSNDQTAGDPLIEHCAKFGGADHLTDVTLESAWAALQSQIKTDSSTNAGKALV
LGAVDSIGGGAFAGCTGNKVTNGGQCVQYKAMHFANGETTISWLSALRRAAEKVKSDEDN
AKKAQQVEKGLQPLKDTVIAILVGEKTTATAALVEESASKKQQE

NCBI blast 2 sequence scores for N-terminal domains excluding n-terminal signal sequences

Tororo v Tbg: 82%
Tororo v Tbb 927: 80%
Tbg v Tbb927: 79%

CLUSTAL W (1.83) multiple sequence alignment

```
Mul4            MTNAKRAFISFKTLFLITTTNLFTAEANTAPGDNTAQYKAICMLVNLANKCTKGGQATTT 60
TbgDal          MTNAQRAFISFKTLFLITTTNLFTAEDNTAPGDNTAQYKAICMLVNLVKKCTKGGQANTT 60
Tbb927          MTNAKRAFIRFKTLFLLTAANLFTAEANTAAGDNTAQYKAICMLVNLATKCTKGGQEATT 60
                ****:**** ******:*::****** ***.****************..*******  **

Mul4            KTDTALVVGAINISLASNDFQQAIDTEKDWATLPQDKKNKLGGSEEDWQFWKESKRQLKK 120
TbgDal          KTDTALVVGAINISLAGSDFQQAIDTEKELATLPQDNKNKLGGNKADWKFWKESKKQLKN 120
Tbb927          KTDTALVVGALNISLAGSDFQQAIDTEKEWATLPQANKNKLGGSEEDWQFWKESKKQLKH 120
                **********:*****..**********: ***** :******.: **:******:***:

Mul4            HQAWIKTFTGQPKTAAQRLATAEMTLRARKLYEQAQSASATPATSTAQLCKDALYGQGKT 180
TbgDal          HQTWIKTFAAQPKTAAKRLATVEMAIRARNLYAEANQASATPAAYAAQLCKEALYGQGQT 180
Tbb927          HQTWIKTFTAQPNTAAQRLATAEMAKRAKKLYAQAQTAEQTPAPTAAQLCKDSLYGHGAA 180
                **:*****:.**:***:****.**: **::** :*: *. ***. :*****::***:* :

Mul4            SSDGLAAGHATRANVCSDEAAGTNNKAGTALFWDVLCLCGGSSTHANTKQACGKGLEASE 240
TbgDal          SSYGLKAGHATRENVCSEEAAGTSNKAGSALFWDVLCLCGGSSTHANTKQACGKGL-ASE 239
Tbb927          NSDGLKAGHSARENVCSEQAGGPNNKAGTALFWDLLCLCAGDGQQETTKKACGKGLEGSE 240
                .* ** ***::* ****::*.*..****:*****:****.*.. : .**:****** .**

Mul4            TRTDWTPNTNDQAAGDPLIQHCATFGGAGHLNAVTLESAWAALQTQIKTDSNTNAGKPLV 300
TbgDal          TSTDWTPNSNDQTAGDPLIEHCAKFGGADHLTDVTLESAWAALQSQIKTDSSTNAGKALV 299
Tbb927          TRTAWAPDGNDKEAGDPLIEHCAKFGGAGHLTAVTLEAAWAALQTQIKTDSSTNAGKALV 300
                * * *:*: **: ******:***.****.**. ****:******:******.*****.**

Mul4            LGAVDASG-AAFTGCTGNKVTNGGQCVQYKAKHFSNGETTIPWLSALRRAAEKAKSDEDN 359
TbgDal          LGAVDSIGGGAFAGCTGNKVTNGGQCVQYKAMHFANGETTISWLSALRRAAEKVKSDEDN 359
Tbb927          LGAVDSVSGGAFAGCTGNKGTNGGQCVQYKPKHFANGETTIPWLTALRRAAEKVKNDEDN 360
                *****: . .**:****** **********. **:******.**:********.*.****

Mul4            AKKAQQVEKELQLLNDTVIAILVGEKTTTTTAASAEESASKKQQE 404
TbgDal          AKKAQQVEKGLQPLKDTVIAILVGEKTTAT-AALVEESASKKQQE 403
Tbb927          AKKAQQVEKELQLLNDTVIAILVGEKTTAT-AAPVEESASKKHQE 404
                ********* ** *:*************:* ** .*******:**
```
